# Evaluation of antenatal Point-of-Care Ultrasound (PoCUS) training: a systematic review

**DOI:** 10.1080/10872981.2022.2041366

**Published:** 2022-04-05

**Authors:** Amber Bidner, Eva Bezak, Nayana Parange

**Affiliations:** a Department of Allied Health and Human Performance, The University of South Australia, Adelaide, South Australia, Australia; b Department of Physics, The University of Adelaide, Adelaide, South Australia, Australia

**Keywords:** Medical education, training, antenatal, obstetrics, Point-of-Care Ultrasound (PoCUS), continuing professional development, low-resource setting, rural, remote

## Abstract

**Introduction:**

There is limited access to life-saving antenatal ultrasound in rural and low-resource settings largely due to shortages in skilled staff. Studies have shown healthcare practitioners can be upskilled in PoCUS through focused training, offering a viable solution to this deficit. However, standards for training and competency assessment are unclear and regulation surrounding practice is lacking. We aimed to review published literature examining antenatal PoCUS training programs, comparing teaching approaches and study methodologies.

**Methods:**

A search of electronic databases EMBASE, MEDLINE and Google Scholar was conducted. Original research articles evaluating antenatal PoCUS training of healthcare professionals worldwide were identified for analysis. Articles with limited detail on the PoCUS training intervention and those describing comprehensive diagnostic training programs were excluded. Evaluations were compared against the Kirkpatrick Evaluation Framework (KEF).

**Results:**

Twenty-seven studies were included from an initial search result of 484 articles. There was considerable heterogeneity between the PoCUS training programs described. Course duration ranged from 3 hours to 2 years, with 11 of the 27 studies delivering obstetric-exclusive content. 44% trained multidisciplinary groups of health professionals. Long-term follow-up training and skills assessments were lacking in over half of the reviewed studies. Study quality and reporting detail varied, but overall beneficial outcomes were reported with 3/4s of the studies reaching upper KEF levels 3 and 4.

**Conclusion:**

PoCUS performed by upskilled healthcare professionals offers an attractive solution to the problem of inequitable access to antenatal ultrasound. A review of available literature highlighted a paucity of comparable high-quality studies needed to establish a stronger evidence base for antenatal PoCUS, and a need to standardise training and competency assessment. This review may inform educators, researchers and policy-makers on existing training formats and methodologies to assist in establishing best practice antenatal PoCUS training methods for safe service delivery by remote healthcare professionals.

## Introduction

Antenatal ultrasound is the primary imaging modality in pregnancy [1,2], routinely used to estimate due dates, monitor fetal growth and well-being, detect anomalies, and guide specialist referral [3,4]. It can also facilitate the early detection of life-threatening complications such as ectopic pregnancy, fetal malpresentation, multiple pregnancies, placenta praevia and placental abruption [5–8]. The International Society of Ultrasound in Obstetrics and Gynaecology (ISUOG) have published guidelines recommending women receive two antenatal US examinations during a normal low-risk pregnancy [9]. However, studies into service accessibility in rural, remote and low-resource settings around the world indicate women are not receiving this care [10–12]. The WHO estimates most maternal deaths are preventable, with over 90% occurring in low-resource settings. Approximately 86% of estimated global maternal deaths in 2017 were attributed to the developing nations of sub-Saharan Africa and Southern Asia [13]. The majority of neonatal mortality also occurred in these regions [14]. A 2015 survey of healthcare providers in South America, Africa and Asia reported the primary reason for not making ultrasound available to pregnant women was a lack of suitable education [15]. Skill shortages are also evident in developed nations like the USA, Canada and Australia, where many remote medical centres have no onsite sonographer and rely on visiting professionals available as infrequently as one day per month [10,16]. The recent COVID-19 pandemic has increased uncertainties in travel and logistics, impacting locum staffing in rural areas and highlighting the importance of trained remote healthcare workers. Accurate estimation of due dates and early detection of potentially life-threatening complications are crucial for remotely located women, who may need days of travel to access specialist obstetric care [7,10,17]. It is in these low-resource settings that antenatal Point-of-Care ultrasound (PoCUS) can offer substantial benefits.

Modern portable ultrasound machines capable of producing high-quality images are affordable and have helped establish PoCUS in many medical fields [1,3]. Performed and interpreted at the bedside by the healthcare provider, PoCUS allows for focused studies to assist procedures or direct care and referal [18]. As a highly skilled, operator-dependent modality, PoCUS requires appropriate and ongoing training of experienced healthcare professionals [3]. It takes years of study and training to produce qualified sonographers, and once trained it is challenging to entice these professionals to relocate and remain in rural locations [16,19,20]. There is growing evidence indicating PoCUS training programs can effectively teach the skills necessary to allow for task-shifting of focussed ultrasound examinations from sonographers to doctors, nurses and midwives [21–26]. Ultrasound training is being increasingly incorporated into undergraduate medical curricula and on the job training [26–28], but it is less well established in non-physician (nursing and midwifery) programs [29,30]. In most developing countries and low-resource settings, antenatal care is provided primarily by midwives and nursing staff, which presents an opportunity to task-shift and upskill these essential workers [21–26].

**Table 1** lists general PoCUS workshop requirements and methods for assessing competency [31–33]. The WHO recommends a standardised curriculum and competency assessment be adopted by all countries for antenatal PoCUS training [5]. However, training guidelines and standards to ensure a minimum level of competency for safe practice vary between countries, with PoCUS remaining largely unregulated globally [34–36]. In many countries, health practitioners may perform PoCUS with little or no training, and without formal accreditation, leading authorities to call for reform and regulation of its use [16,37]. Ultrasound performed by untrained clinicians may represent a higher risk of misdiagnoses. Overlooked health conditions may lead to delayed diagnoses and treatment (‘false negatives’), while misinterpretations and incidental findings (‘false positives’) can cause considerable patient anxiety and unnecessary follow-up investigations, increasing the economic burden on the healthcare system [38–40].

**Table 1.** General PoCUS workshop requirements and methods for assessing competence

General workshop requirements defined by *ASUM[31,32]	Methods for assessing competency in PoCUS[33]
Faculty- must include a medical specialist with appropriate and extensive clinical experience/qualifications. Instructors must have significant practical experience in the application being taught. Registered sonographers can assist with teaching skills.	Technical competency assessment- Probe selection, image mode selection (e.g., cardiac, obstetric), proper image orientation, probe positioning, depth, gain, centering of target structure, demonstrates advanced functions (M-mode, Doppler, image capture), troubleshooting.
Teaching (including practical) hours should at least meet those published in credentialing syllabus for the application taught.	Knowledge assessment- Course entry assessment following pre-reading Pre and post course assessment Multiple-choice questions Written answer
Provision of course syllabus, learning materials, recommended texts and other references.	Objective Structured Clinical Examination (OSCE) / Practical examination.
Instructor to candidate ratio 1:5.	Standardised checklists for evaluating technical skill.
Machine to candidate ratio 1:5.	Skill assessment on simulator, model, or standardised patient.
Appropriate models and patients.	Review of images obtained on real patients.
Setting to accommodate lectures and practical scanning sessions.	Real-time assessment of scanning actual patients and clinical decision making.
Pre- and post-course tests.	Longitudinal patient evaluation and periodic review to assess image quality and accuracy of PoCUS interpretation.
Evidence of attendance including course hours.	Self-assessment- Knowledge/skill Perceptions/attitudes Change in work behaviour/scanning frequency

*ASUM- Australasian Society for Ultrasound in Medicine.

Endorsement for outreach training programs have been provided by the Australasian Society for Ultrasound in Medicine (ASUM), ISUOG, World Federation for Ultrasound in Medicine and Biology and RAD-AID, indicating a global effort to address the skill shortage [35]. This literature review examines the training and evaluation methods being employed to teach antenatal PoCUS to medical and allied health professionals.

## Methods

This review investigated international literature on antenatal PoCUS education from 2000 to January 2021, focusing on publications that evaluate the efficacy of training models. It has adopted the ‘Preferred Reporting Items for Systematic Reviews and Meta-analysis’ (PRISMA) guidelines and was formerly registered with the international prospective register of systematic reviews (PROSPERO), registration number CRD42021230267.

A team of three researchers from the University of South Australia with experience in research, tertiary education and clinical practice (including ultrasound and PoCUS training in low-resource settings) conducted the review and reached consensus on the eligibility criteria, search strategy and terms, final article inclusion, data extraction and quality assessment.

### Search strategy

A systematic search of electronic databases EMBASE and MEDLINE was conducted through OVID for original research literature, performed on the 7^th^ of January 2021. An experienced librarian was consulted to assist in the design of search terms and strings, which were then reviewed by all members of the research team. The search was limited to ‘Human’ and ‘English’ language only. No restrictions were set regarding the publication year. Search terms used for both databases were grouped into main four areas and combined using terms synonymous with pregnancy, ultrasound, point-of-care, and training. A grey literature search was conducted through Google Scholar (5 pages, 50 results) using the key search terms (pregnancy/antenatal, point-of-care ultrasound/ultrasound, and training/education). Connected papers (https://www.connectedpapers.com/) was searched to canvas for additional relevant articles (see **Appendix Table A1: Search strategy**).

### Eligibility criteria

For inclusion, an original research study must have described and evaluated an ultrasound training program intended for point-of-care or bedside application on antenatal patients. Articles involving PoCUS obstetric training as part of a broader training curriculum were eligible. All medical and allied health specialties were included as the training participant population, and pre-graduate students from all health disciplines. No restrictions were placed on study/training setting.

Studies evaluating advanced training (complex and interventional scanning) or formal diagnostic ultrasound programs leading to qualification as a sonographer were excluded. Articles with limited descriptions of the training program provided were excluded, as were conference abstracts/reviews, editorials, commentaries and letters (see **Table 2: Eligibility criteria**).

**Table 2.** Eligibility criteria

	Include	Exclude	Rationale for exclusion
Population	Health care clinicians/practitioners- Nurses, Midwives, Doctors, Allied health workers, students from all health disciplines.	Sonographers Non-health professionals.	Sonographers and trainee sonographers possess more advanced imaging skills and are not within the scope of this review. Focus on training of populations with minimum basic healthcare training/experience/qualification. It is not advisable for non-healthcare professionals without training to perform PoCUS on patients/people.
Intervention/ Exposure	Antenatal Point-of-Care ultrasound (PoCUS) training including broader courses teaching scanning of multiple organ systems.	PoCUS training with no antenatal specific content (e.g., critical care- FAST, Abdominal, cardiac, lung, vascular). Advanced training (complex and interventional scanning) or full diagnostic ultrasound training leading to registration and qualification (sonographer training).	Non-antenatal PoCUS training is beyond the scope of this review. This review is focused on basic PoCUS training of health professionals. Advanced training such as those requiring lengthy courses, long-term supervision and formal accreditation/registration are beyond the scope of this review.
Outcome	Efficacy of training.Types of evidence/evaluation measures: Training course evaluation/Trainee satisfaction, testing of knowledge and practical skills (OSCE), Image quality review, diagnosis review, Confidence measures, Scanning frequency, Maternal/fetal outcomes.	Articles with limited description or reporting of the training intervention (training delivered), training assessment (evaluation measures) and outcome.	Inadequate methodological detail inhibits quality assessment and comparison between studies.
Study type	Original research. Studies involving antenatal PoCUS training evaluation- Cohort studies, cross-sectional studies, case control studies, observational studies, qualitative studies, case reports, clinical and randomized control trials. English language. Clinical/Human studies.	Review articles, Conference presentations/Abstracts, Letters, Editorials, Commentaries.Articles with Insufficient detail of described training interventions (training delivered), training assessment (evaluation measures) and outcome. Non-English articles. Animal studies.	Only original research articles were included for review. Conference presentations and abstracts (not full length articles) provide insufficient detail and peer-review scrutiny.

The database search was performed by the primary author. Duplicates were removed prior to the initial title and abstract screening conducted independently by two reviewers, who then performed full-text reviews. Non-consensus at both initial title/abstract screening and later full-text screening was decided by a third independent reviewer. The citations of all identified articles included in the review were searched, with title/abstract then full-text screening performed by two independent reviewers.

### Data extraction and synthesis

All three reviewers discussed themes and agreed on data points to be extracted, and reviewed these pre-determined categories in a Microsoft Excel spreadsheet prior to data extraction. Data was extracted to the spreadsheet and collated by the primary author and examined by a second reviewer. Meta-analysis was not possible due to the heterogeneous teaching and assessment methodologies used, thus an integrative approach to data synthesis was employed. Emerging themes were discussed amongst all three reviewers and a narrative response was composed. Comprehensive tables summarising the reviewed studies’ training and evaluation methods, and key investigated outcomes are included to facilitate comparison.

### Quality assessment

A modified Medical Education Research Quality Index (MERSQI) tool (see **Appendix Table A2**) was used to assess the quality of the included articles. The MERSQI is a validated assessment instrument used in medical education research to measure the quality of experimental, quasi-experimental and observational studies [41,42]. The tool was modified to include two categories: 1) ‘Number of trainees’ (score of 0.5 to 1.5), and 2) ‘Follow-up of training’ (score of 0 to 3). These domains were considered valuable to this review given the variation of recruited participant numbers between studies (impacting study power), and the importance of follow-up training, assessment and ongoing support of trainees for learning PoCUS and ensuring safe practice. The percentage response rate was omitted as this measure was not applicable to the vast majority of the studies. Each domain was scored out of 3. The primary author performed the quality assessment, grading all articles within a potential score range of 5 to 21. A risk of bias assessment was also conducted relating to five key areas of educational development: Underpinning bias, Resource bias, Setting bias, Content bias, Educational/Development bias (see **Appendix Table A3: Risk of bias assessment tool**). However, assessment of quality and risk of bias did not restrict article inclusion in this review’s final synthesis, ensuring the inclusion of a broad cross-section of literature representative of the range of study quality and methodologies in published circulation.

Each study reviewed was compared against The Kirkpatrick Evaluation Framework (KEF), a validated four level model designed to evaluate and classify training and development programs [43,44]. The four levels described (Level 1- Reaction; Level 2- Learning; Level 3- Behaviour; Level 4- Results) by the Kirkpatrick model represent a continuum of complexity and value in evaluation measures. Level 4, representing the highest evaluation measure (healthcare or patient-related outcomes), assesses impact and aligns with the MERSQI ‘Outcomes’ domain.

## Results

**Figure 1.**
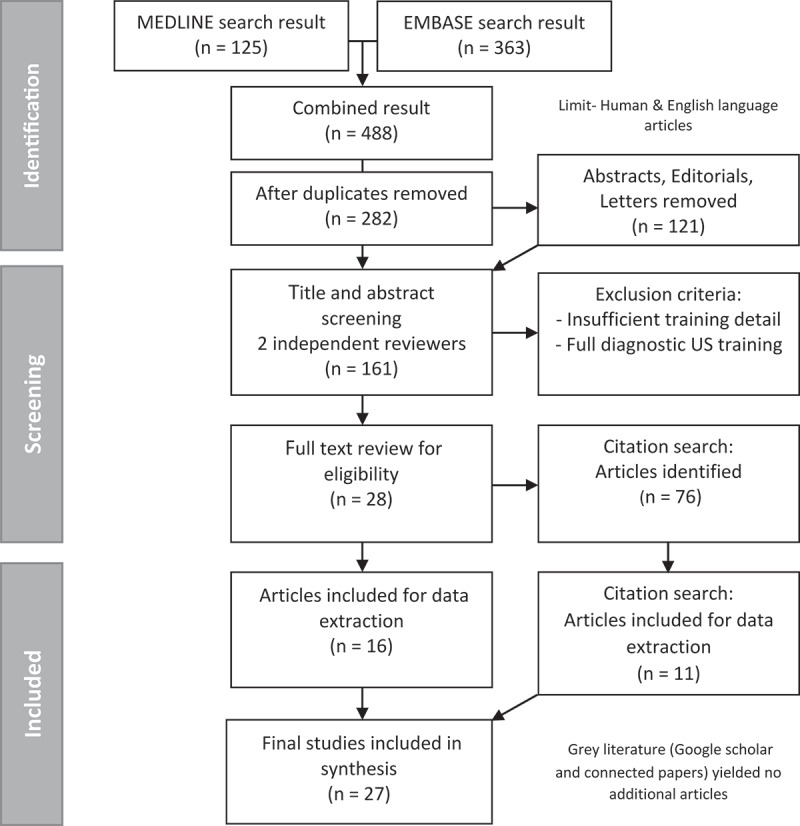
PRISMA framework – Search results.

Twenty-seven studies were included, from an initial 484 articles retrieved in the OVID database search during the identification stage (see Figure 1**: PRISMA framework**). Of the 27 articles, 16 were identified through EMBASE and MEDLINE, with a further 11 obtained through citation searches. Google Scholar and Connected papers searches yield no additional eligible articles.

The identified studies spanned two decades; the oldest study published in the year 2000. Three-quarters were published after 2014 reflecting the growing interest in PoCUS research. Most were single arm interventional studies using convenience samples of health professionals and patients. Four of the studies were conducted by the same research team, Shah et al. [45–48]. Twenty studies were conducted in developing countries, of which only five were conducted in urban settings. Seven studies included developed countries (five in the USA, all urban areas with one intended for rural deployment; one in Australia and one in Denmark). Two of these studies used training sites in both developed and developing countries; one [49] employed a clustered randomised control trial design that was carried out over five clinical sites located in Africa, Asia and America, and the other [50] compared six training sites and formats conducted in rural and urban settings in Australia, Timor Leste and Indonesia. Key outcomes investigated and the main findings of the studies are summarised in **Table 3**.

**Table 3.** Key outcomes investigated and findings

First author Year Published	Key outcomes investigated		Main Finding in relation to antenatal PoCUS	
Adler [66] 2008	Frequency & application of PoCUS following training.		Pregnancy-related exams accounted for 24.1% of total. US is a feasible & sustainable imaging modality in a very low-resource setting (refugee camp).	
Baltarowich [61] 2009	Trainee knowledge acquisition & retention – written test scores compared at 3 points in time over 6 months.		Mean test scores increased significantly- 58.4% at entry to 76.8% at end-of-program. Nine of the 12 physicians were selected to establish affiliated US training programs in their own country.	
Bell [22] 2016	Written & practical test scores; before & after refresher training. Frequency & application of PoCUS following training. PoCUS impact on patient management.		Strong correlation between knowledge & practical skill scores. Increase proportion passing both knowledge & practical tests at follow-up, compared to initial session. 90% trainees completing more than 1 session maintained or improved scores. Follow-up survey- 2/3 to 3/4 reported using PoCUS over 20 times in previous 3 months, Obstetrical exams were most commonly performed and had the greatest impact on patient management.	
Dalmacion [25] 2018	Trainee to instructor image comparison. Estimate of maternal/fetal deaths averted.		95% agreement between the trainee & instructor US. Estimated 6.3% of maternal deaths & 14.6% of neonatal deaths possibly averted by the early US screening.	
Dornhofer [54] 2020	Pre- & post-course written & practical examination scores (compare physicians, nurses, & midwives). Post-course survey of trainee scanning confidence.		No participants passed (>65%) the pre-course examination. 43 (72%) passed the course & 12 (28%) failed. Written exam pass rate- Fifteen (79%) physicians, 11 (85%) nurses, & 13 (68%) midwives. Average practical examination score was 83% (SD = 16.2%) for physicians, 82% (SD = 11.4%) for nurses, and 71% (SD = 15.2%) for midwives. Physicians performed best on pre- & post course written exams, followed by nurses & then midwives. Nurses scores improved most, followed by physicians & midwives. The average practical score for physicians was significantly greater than midwives (P = .038). The average practical score for nurses was significantly greater than for midwives (P = .049). All trainees reported increased post course comfort levels performing PoCUS.	
Henwood [53] 2017	Expert review of trainee images. PoCUS application. PoCUS impact on patient management.		100% sensitivity & 98% specificity for expert reviewed OB images (94% & 98% overall). Trainees most frequently used US for abdominal & OB applications. 81.3% of patients had at least one clinical decision changed because of a PoCUS.	
Kimberly [62] 2010	Practical assessment (OSCE) of skills & sustainability over time (2 & 6 months). Expert review of trainee images. Frequency & application of PoCUS following training. PoCUS impact on clinical decision making/patient management.		Paired OSCE scores- slight overall improvement over time. Trainees most competent at identifying number of gestations (100%), & fetal presentation (96%), calculating FHR (48%) was more challenging. Scan review- FHR interpretation 96% agreement, placental location 91% agreement, BPD 70% agreement. Mean of 21 scans performed per trainee over 6 months. 2^nd^ & 3^rd^ trimester most common. Main indications recorded- Size to date discrepancy (44%) & determining fetal position (39%). US prompted change in clinical decision-making in 17% of cases. At 1 year follow-up- trainees average 10 PoCUS per week. 85% reported helping colleagues use US. 46% reported significant time constraints as main limitation.	
Kolbe [26] 2015	Expert review of trainee images. PoCUS application/indication. PoCUS impact on clinical decision making/Patent management.		Average expert rating of trainee images-6.54/10 in first 6 weeks & 7.17/10 in last 6 weeks. 52% (CI- 44-61%) of patients had a new diagnosis after PoCUS. A new diagnosis lead to change in management in 48% (CI 40–57%) of patients.	
Kotagal [56] 2015	Change in self-assessment scores regarding attitudes, confidence & assessment of the value of US before & after training intervention.		Mean confidence score pre-test to post-test improved from 23.3 (±10.2) to 37.8 (±6.7). Before & after training, trainees overwhelmingly agreed US would improve their practice, make them a better surgical resident, & improve their practice in LRS. All agreed the US course helped them improve their PoCUS knowledge & skills.	
Lathrop [58] 2011	Learner portfolio & hands-on workshop for US credentialing & training. Learner feedback meeting.		All 4 trainees credentialed to perform US clinically within one month. Portfolios & evaluation rubric offered a consistent, systematic means to demonstrate the acquisition of skills for clinical practice. And were a more effective & practical method of demonstrating trainee competence & supporting credentialing over a physician’s subjective impression of trainees’ abilities.	
Lee [55] 2017	Pre & post course trainee knowledge assessment. Post training practical skills assessment and course evaluation survey (scanning confidence & intent-to-use US).		Average pre-course exam score was 35.2% (2.4% pass rate). The average post-course exam score was 82.0% (92.7% pass rate). Average practical score on completion of the course was 83.2% (*SD *= 0.145) with 82.9% of the class passing (pass mark above 75.0%). Post-course survey- overall increased level of comfort performing all scans. Cardiac followed by OB US were anticipated to be most frequent indication.	
Lindgaard [52] 2017	Expert review of trainee images.		Expert to trainee agreement for intrauterine pregnancy-100%, GA- 93%. Low-to-moderate complexity PoCUS exams performed by GPs with sufficient prior training have a very high level of inter-rater agreement when compared to exams conducted by radiologists & gynaecologists.	
Mandavia [67] 2000	Trainee knowledge acquisition & retention- written test scores before & after training & at 10 month follow-up (stratified by discipline & US experience). Expert review of trainee images. Frequency & application of PoCUS.		The mean pre-test score was 65%, mean post-test score 84%, No decline after 10 months. Pre-test variation based on US experience not evident after training. Image review- overall sensitivity of 92.4% & specificity of 96.1% (95% CI = 94% to 98%). OB only exams were 94% sensitive & 100% specific. Frequency of scanning varied widely (9–152), averaging 62 exams over 10 months. Biliary, renal and trauma were the most frequent indications for scanning.	
Nathan [49] 2017	Written & practical skills assessment. Expert review of trainee images for errors in scanning parameters & diagnosis, using predetermined criteria. Site, patient & trainee demographics.		36/41 trainees passed the practical test on first attempt at the end of 2 week course & 40/41 passed at the end of 12 week pilot period. Mean practical skills score increased- 78% on the first test to 92% on the fourth test. Of the 3801 US exams (32,480 images), 94.8% were rated as satisfactory by expert review. Concordance between trainee & reviewer US diagnosis was 99.4%. High-risk pregnancies were identified by the trainees in 6.7% (255/3801) of exams.	
Rominger [24] 2018	Frequency & application of PoCUS following training. Expert review of trainee images. Impact on patient management & diagnosis.		The most common studies were TA OB exams (45.5%) and abdomen/pelvis (26.6%). US scanning peaked after teaching sessions then gradually decreased over months. Highest recorded scans were after the final teaching session. Disagreement in findings in 4.3% of the images reviewed (none affected clinical management) & 6.5% with inadequate image quality to interpret. PoCUS changed patient diagnosis in 34% (24% for OB patients) & clinical management in 30% (20% for OB patients). In the scans that changed the diagnosis, 78% led to changed clinical management.	
Shah [45] 2020	Practical OSCE assessment & accuracy of images & measures over time. Blinded expert image review for QA & inter-rater reliability of reviewers. Confidence levels pre-post training & at 3 months follow up. Post course evaluation/training perception interviews.		Of 25 trainees, 22 passed (average score 89.4%) the OSCE on first attempt. Image quality improved with time; the final error rate at week 8 was less than 5%. Confidence levels increased- pre-course 1 point average to over 6 points post-course for all measures (maximum 7). Key informant interviews- indicated a desire for more hands-on training, longer training duration & challenges in balancing clinical duties with ability to attend training sessions.	
Shah [48] 2014	Pre- & post-course knowledge & confidence assessment. Time & accuracy of scanning.		For previously untrained trainees, pre- & post-test knowledge scores improved from 65.7% [SD = 20.8] to 90% [SD = 8.2] (p < 0.0007). Self-confidence improved significantly for identification of FHR, fetal lie, & EGA. Average times for completion of critical skills: cardiac activity (9s), FHR (68.6s), fetal lie (28.1s), & EGA (158.1 sec). EGA estimates averaged 28w0d (25w0d-30w0d) for the model‘s true GA of 27w0d.	
Shah [47] 2009	Reporting impact of previously published training program (Shah 2008) Patient demographics & US application. Blinded expert image review of trainee images for quality & accuracy. PoCUS directed change of patient management.		OB scanning was the most frequently used application followed by abdominal. Evaluation of GA, fetal head position, & placental positioning were the most common findings. Local staff performed 245 US scans in the 11 weeks after the departure of the US instructor. Expert to trainee agreement on scan review of 96%. US changed patient management in 43% of patients.	
Shah [46] 2008	Initial US needs assessment, training curriculum development & implementation. Staff survey of prior US experience & hospital records review.		10 of 15 physicians completed the training. Needs assessment-all 15 trainees rated OBs most important application. Focus group discussion- barriers to US services included distance, time & cost for transfers, lack of monitoring during transfers & US charges. Dissatisfaction expressed US report quality & inability to view images with written reports.	
Shaw-Battista [29] 2015	Pre & Post course knowledge assessment. Post course evaluation. Other project evaluations/outcomes ongoing & not reported including: pre- & post-online module knowledge of OB US, interprofessional competencies, & post-training knowledge following seminar & practical.		Course evaluations were extremely positive. Trainees expressed enthusiasm to develop basic US competencies & recognised the applicability of new skills to clinical practice. Hands-on sessions were universally appreciated- requests for additional or longer sessions, more pregnant volunteers & reducing group size (trainee to faculty ratios). Trainees reported teaching varied types of trainees together as “innovative & helpful” but also perceived to be challenging.	
Shokoohi [51] 2019	Trainee demographics including US experience. Frequency & application of PoCUS following training. Teaching of other staff/students. Challenges/barriers to integrating US into patient care.		Main applications for PoCUS- cardiac exams followed by 2nd & 3rd trimester OB exams. Over 75% reported use of PoCUS in clinical diagnoses & 50% in determining treatment. 50% reported very frequently or often using US to teach within their clinics. Largest perceived barriers- lack of clinical educators US knowledge, lack of time, equipment security, difficulty accessing the Internet & equipment problems.	
Stolz [30] 2015	Frequency & application of PoCUS over duration of training course. Patient/scan outcome.		Of 22,639 ED patients evaluated, PoCUS examinations were performed on 1,886 patients. OB scans (9.3%) were 3rd most common scan after FAST (53.3%) & Echo (16.4%). PoCUS studies were performed more frequently than radiology department-performed studies. Positive findings were documented in 46% of all PoCUS exams.	
Swanson [85] 2014	Expert review of trainee images. Diagnostic outcome following PoCUS.		Expert review of trainee images- 100% sensitivity & specificity for identifying gestational number, 90% sensitivity & 96% specificity for fetal presentation. Trainee PoCUS altered clinical diagnosis in up to 12% of clinical encounters.	
Vinayak [63] 2017	Post e-module assessment (pre hands on) & post course written assessment. Expert review of trainee images for quality & accuracy of interpretation. Patient outcome following PoCUS. Patient experience.		E-module knowledge reported useful. All trainees passed the written post-course exit exam on 1st attempt. Reporting accuracy of trainees’ scans was 99.63%. Reduced AFI missed on 2 patients scans. Time to complete scan halved after completing 30 scans. All 246 patients felt the process was safe, convenient & reassuring, had a better antenatal visit experience & increased confidence in care delivery. More spouses attended then for routine antenatal visits.	
Vyas [70] 2018	Post-training OSCE practical assessment. Image quality review & trainee identification of pathology. Patient outcome.		Trainees were able to correctly identify fetal presentation, fetal number, & placental position in all enrolled patients. BPD correctly assessed in 95.3% & HC 90%. GA had a mean difference from expert sonographers of 1.5 days (BPD) & 0.26 days (HC). All 4 patients with abnormal findings were expert confirmed.	
Wanjiku [21] 2018	Pre-training knowledge assessment. OSCE practical assessment with image quality scores. Frequency & application of PoCUS following training.		OB images received the highest mean image quality score (compare to FAST, thoracic & echocardiography). Image quality scores increased with an increase in training sessions and decreased with increasing time since prior training. OB US were most frequently performed. Frequency of scanning positively correlated with written & image quality test scores.	
Westerway [50] 2019	Trainee knowledge acquisition & retention – written & practical tests before & after training & at 6/11 month follow-up.Comparison of PoCUS courses (rural/urban sites, duration, student numbers).Course evaluation- satisfaction, engagement, understanding & relevance of learning. Scanning on return to work.		Practical assessment at 6/11 months- minor prompting for image optimisation (depth, gain & imaging plane for fetal biometry) for all but 4 trainees who had peer support at work following training. All (55) trainees achieved the course objectives, regardless of format. Course evaluation- all trainees stated understanding what was taught & relevance to their clinical work. All trainees continued scanning on return to work.	
**Abbreviations** AFI- amniotic fluid index BPD- Biparietal diameter CI- Confidence interval d- days ED – Emergency Department EGA- Estimated gestational age		FAST- Focused assessment with sonography in trauma FHR- Fetal heart rate GA- Gestational age HC- Head circumference LRS- Low-Resource-Setting OB- Obstetric		OSCE- Objective Structured Clinical Examination PoCUS- Point-of-Care Ultrasound QA- Quality assurance SD- Standard deviation US- Ultrasound w- weeks

## Training methods and delivery

A summary of the included studies’ teaching methods, including duration and location, trainee and instructor demographics, curriculum and practical skills taught, and follow-up training/support, is provided in **Appendix Table A4: Training methods and delivery**.

### Curricula

**Figure 2.**
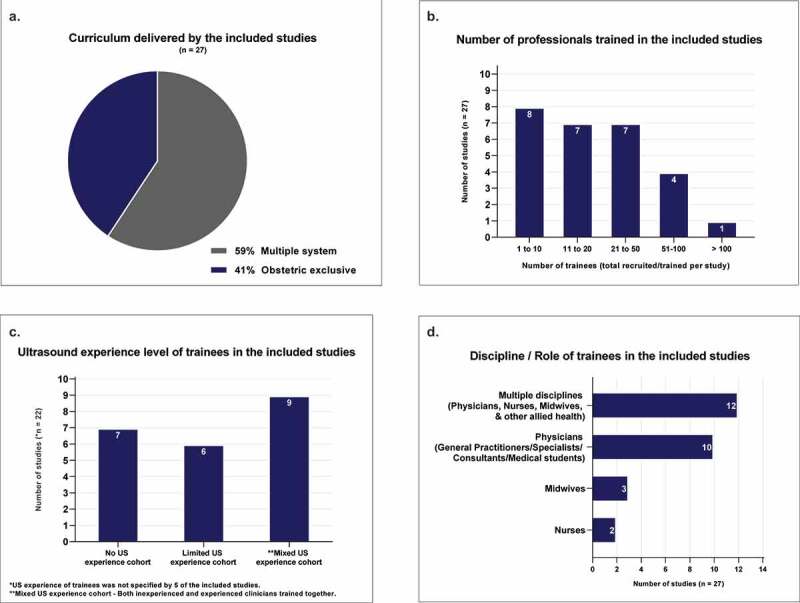
Of the included studies in the review a. Curriculum delivered (Multiple organ systems taught or Obstetrics and gynaecology only); b. Number of professionals trained (total recruited and trained per study); c. Ultrasound experience of trainees prior to undertaking the training; d. Discipline/role of the trainees (Multidiscipline/Physicians/Nurses/Midwives).

Course duration ranged from 3 hours to 2 years. Obstetric-exclusive curricula were delivered in 11 of the 27 studies (see Figure 2a). Of the studies that covered multiple-organ systems, several delivered their content in as few as 1–2 days [21,22,51,52], with either refresher sessions or additional online learning modules provided to support the intensive practical training. When designing intensive courses with limited delivery time, other resources to supplement face-to-face training, such as digital and written learning modules/resources, and online/distance teaching and feedback are important. Many of the studies employed these supportive measures (see **Appendix Table A4**). Maintaining a focussed curriculum for specific predefined clinical indications and remote technical assistance was recommended [51].

The course topics and practical skills taught by each study are listed in **Table A4** of the **Appendix**; these ranged from basic assessments like fetal lie/presentation to more complex fetal biometry, heart assessment and the detection of anomalies such as ventriculomegaly, anencephaly, hydronephrosis, and spina bifida. A training needs assessment was conducted by five of the studies [24,26,46,47,53] which informed the choice of ultrasound applications that were most relevant for program inclusion. Specific skills were taught if relevant to endemic needs; for example, three studies [51,54,55] conducted in Tuberculosis prevalent regions included ultrasound assessment of HIV/Tuberculosis (FASH) in their multisystem curriculum.

Over two-thirds of the studies detailed ultrasound physics and instrumentation in their curriculum, and four specifically mentioned *‘safety’* [30,45,49,56], consistent with the WHO’s recommendations that trainees are able to monitor mechanical and thermal indices on equipment and understand safety concepts underpinning ultrasound exposure of the patient and fetus [57]. Trainees should understand the limitations of their focussed training and seek assistance with image interpretation and patient management decisions from an experienced sonographer and/or clinician where necessary. Appropriate documentation of the PoCUS exams findings and patient referral is also important. Three of the studies listed ‘documentation’ in their curriculum [29,30,58], one specifically including ‘*liability and risk reduction strategies*’ [29].

### Trainees’ previous ultrasound experience

Participant trainee numbers in any single study ranged from 3 to 162, with a total of 903 trainees across all included studies. Over half of the studies trained fewer than 20 trainees, limiting study power (see Figure 2b). Of the studies that reported trainees’ prior ultrasound experience, nine trained participants with mixed ultrasound experience, six enrolled trainees with limited but similar ultrasound exposure and seven restricted eligibility to trainees with no prior ultrasound training or experience (see Figure 2c). Delivering a program to suit trainees with varied ultrasound education and proficiency was an acknowledged challenge [22,51]. Shokoohi et al. [51] raised concerns that more experienced trainees may not have received the same benefit from their curriculum, particularly in the introductory session. Grouping trainees and designing specific curricula and objectives catered to each groups’ experience and skill level could mitigate this problem.

### Multidisciplinary training groups

The future of healthcare and its education is moving towards a more cooperative interdisciplinary culture [59], demonstrated in the multidisciplinary participant groups trained together in almost half of the reviewed studies. Ten (33%) studies exclusively trained medical physicians and only 5 (19%) were dedicated to nurses and midwives (see Figure 2d). Several authors [29,50] described unique challenges to interprofessional teaching, where individual needs and preferences can vary significantly between trainees. This scenario, however, provides the opportunity to use trainees’ unique experiences and individual strengths to enhance course design and foster collaborative practice. Shaw-Battista et.al [29]. reported enhanced inter-professional collaboration as a benefit of their multidisciplinary PoCUS training initiative, stating it fostered better communication, coordination of care and understanding of other professionals overlapping and unique scopes of practice. Providing clear objectives, varying curricula in breakout groups tailored to participant’s experience level, and the opportunity for peer-led teaching in mixed skills/experience groups would benefit a multidisciplinary PoCUS training cohort.

### Instructors’ experience

Instructor-to-trainee ratios were reported in over half of the studies, mostly for practical training sessions, and ranged from 1:1 to 1:6; guidelines have recommended a maximum ratio of 1:5 [32,60]. The instructors’ experience ranged from second year medical students (aimed at providing low-cost outreach training in developing countries [54,55]) to trained sonographers and medical specialists. Some courses benefited from a multidisciplinary teaching team with specialised content delivered by experts in their field (cardiac, paediatric and obstetric specialists). Several articles included principles of teaching in their curriculum [45,61], and reported scenarios where trainees went on to teach ultrasound to colleagues on return to work [51,61,62]. Training the trainer initiatives, where trainees are taught teaching methods to pass on learning to colleagues, is a useful and potentially cost-saving option in settings with limited resources and access to training. Care should be taken using this teaching model with PoCUS courses of short duration, given the complexities of learning ultrasound and the often unsupervised work environments trainees return to. Instructor qualifications and teaching ratios are provided in **Appendix Table A4**.

### Ethical considerations

Few papers mentioned the ethics surrounding the use of pregnant patients for ultrasound training purposes. Ideally, practical training would utilise both simulated and real-life patients with strictly limited times placed on scanning pregnant volunteers and a heavier reliance on phantom models and virtual/simulation technologies in early training [50,63]. In most studies, only healthy models were used during training, which precludes the demonstration of pathology [29,55]. Simulation can be beneficial in this respect and offers the advantage of learning in a safe, patient-free environment. They have a particular utility in obstetric ultrasound training, but the cost of implementing high-fidelity simulated systems would be prohibitive in the majority of settings reviewed [64,65]. Westerway [50] reported using commercial and handmade phantoms and Vinayak et al. [63] used ‘scanning phantoms’ in the initial week of training, but no high-fidelity simulation systems were used in any of the reviewed studies. Shaw-Battista et al. [29] discussed the ethics of using pregnant models, stating their intention to introduce equipment to simulate first-trimester ultrasound in their next course iteration. This would reduce reliance on pregnant volunteers and provide the opportunity to scan simulated first trimester pregnancies, commonly lacking in training courses due to the early gestation of the fetus and risk of identifying an unexpected abnormality in pregnant volunteers who are yet to receive formal scanning.

### Follow-up training and support

Follow-up training is beneficial to reinforce learning and provides the opportunity to assess knowledge retention, which is important for continued safe practice. Follow-up training sessions, in either face-to-face or online format, were offered by half the studies, with periods varying from 3 months to 2 years. **Appendix Table A4** summarises the follow-up training and support provided by the reviewed studies. Of those who did not report/provide additional follow-up training sessions, five offered assistance through personal telecommunications feedback and image review. Telemedicine was investigated by Kolbe et al. [26] who provided remote real-time scanning and image review. Vinayak et al. [63] used an asynchronous method, where trainee images and interim report was sent for specialist review while the patient waited. Other studies used telecommunications via email and various messaging platforms to remotely assist trainees, provide feedback and review images, but only Kolbe et al. [26] used remote real-time scanning supervision.

## Training evaluation and assessment methods

The approach and method used to evaluate the courses and trainees varied widely across studies. A summary of the evaluation methods reported including knowledge and practical assessment, expert image review, frequency and application of scanning, patient outcomes and trainee feedback/survey is provided in **Table 4: Trainee & course evaluation.**

**Table 4.** Trainee & course evaluation

First author Year Published	Trainee feedback/survey Course evaluation, Scanning confidence, Training needs assessment, Patient feedback	Pre/post course theoretical knowledge assessment	Practical/OSCE assessment	Expert image review	Frequency and/or application of PoCUS	Patient outcomes & management
Adler [66] 2008	**X**	**X**	Minimum 20 supervised US examinations. No structured OSCE or knowledge exam.	**X**	Frequency & application of PoCUS following training.	**X**
Baltarowich [61] 2009	**X**	Identical test at program entry, course completion & 6 months follow-up.	**X**	**X**	**X**	**X**
Bell [22] 2016	**X**	Pre course test- open book 90% pass mark for enrolment.	OSCE- assessing image interpretation & quality.	**X**	Follow-up survey of US use.	PoCUS impact on patient management.
Dalmacion [25] 2018	**X**	Pre & post Knowledge test.	**X**	Expert review of trainee images.	**X**	Estimate of maternal/fetal deaths averted following PoCUS.
Dornhofer [54] 2020	Post course survey to assess scanning confidence & provide course feedback.	Identical pre & post course knowledge test.	Post course practical test on image acquisition & interpretation.	**X**	**X**	**X**
Henwood [53] 2017	Pre-training needs assessment.	**X**	Image based assessment & post course OSCE. Regular practical assessment over 6 month follow-up.	Expert review of trainee images.	Frequency & application of PoCUS following training.	PoCUS impact on clinical decision making/patient management.
Kimberly [62] 2010	**X**	**X**	Practical assessment (14 item OSCE) of skills & sustainability over time (2 & 6 months).	Expert review of trainee images.	Frequency & application of PoCUS following training & teaching colleagues.	PoCUS impact on clinical decision making/patient management.
Kolbe [26] 2015	Pre-training needs assessment.	**X**	**X**	Expert review of trainee images.	Application of post training PoCUS.	Change in patient diagnosis & management following PoCUS.
Kotagal [56] 2015	Pre & post survey to measure trainee confidence.	**X**	**X**	**X**	**X**	**X**
Lathrop [58] 2011	**X**	**X**	**X**	Learner portfolio & images reviewed using rubric to evaluate progress, knowledge & skills prior to credentialing.	**X**	**X**
Lee [55] 2017	Post course evaluation survey.	Identical pre & post knowledge test. Follow-up testing scheduled in 12 months.	OSCE practical assessment.	**X**	**X**	**X**
Lindgaard [52] 2017	**X**	**X**	Short practical assessment.	25 specific US exams (video sequences & screen shots) uploaded for instructor review.	**X**	**X**
Mandavia [67] 2000	**X**	Identical knowledge assessment pre & post course & at 10 month follow-up (24 positive, negative, & nondiagnostic US images for interpretation).	**X**	Expert review of trainee images- sensitivity & specificity.	Frequency & application of PoCUS.	**X**
Nathan [49] 2017	**X**	Written exam at end of 2 week course for pilot eligibility. Monthly practical assessment for 3 months.	Practical exam at end of 2 week course for pilot eligibility.	Expert review of trainee images for errors in scanning parameters & diagnosis, using predetermined criteria.	**X**	Patient outcome- High risk pregnancies.
Rominger[24] 2018	Pre-training needs assessment.	**X**	**X**	Case logs & images (35%) reviewed for quality assurance & feedback.	Frequency & application of PoCUS following training. Scanning frequency over time.	PoCUS impact on patient management & diagnosis.
Shah [45] 2020	Pre & post US confidence survey & at 3 month follow-up. Key informant interviews assessed trainees’ perception of training program.	**X**	25 proctored scans prior to final OSCE (80% pass mark).	2 months of blinded expert image review & inter-rater reliability of trainee scans.	**X**	**X**
Shah [48] 2014	Post course confidence survey	Pre and post course knowledge assessment.	Time & accuracy of scanning recorded during practical training.	**X**	**X**	**X**
Shah [47] 2009	Pre-training needs assessment.	**X**	**X**	Expert review of trainee images for quality & accuracy of interpretation.	**X**	PoCUS directed change of patient management.
Shah [46] 2008	Pre-training needs assessment. Focus group discussion of barriers to US utilisation.	**X**	**X**	**X**	**X**	**X**
Shaw-Battista [29] 2015	Post course evaluation immediately following practical training.	Pre & post online module knowledge test. Passing grade required before seminar & practical training. Post-practical training knowledge test (results not reported).	**X**	**X**	**X**	**X**
Shokoohi [51] 2019	Post training survey to evaluate course, trainee demographics, medical/PoCUS experience, US use, challenges/barriers & opinions.	**X**	**X**	**X**	Frequency & application of US (including teaching others)	**X**
Stolz [30] 2015	**X**	Invigilated examinations conducted throughout 2 year training period.	Invigilated examinations conducted throughout 2 year training period.	**X**	Frequency & application of PoCUS over duration of training course.	Patient outcome following PoCUS.
Swanson [85] 2014	**X**	Oral competency tests on return to clinic following training.	Practical competency tests on return to clinic following training.	Expert review of trainee images- Sensitivity & specificity for clinical indications.	**X**	Patient outcome/ Altered diagnosis following trainee PoCUS.
Vinayak [63] 2017	Patient survey of experience following trainee provided PoCUS.	Pre-test following e-module. Pass mark 100% within 5 attempts to progress to practical training. Post course written assessment.	Practical assessment throughout the course. Post course practical assessment.	Expert review of trainee images for quality & accuracy of interpretation.	Time to complete PoCUS & improvement with time.	Patient outcomes (high risk pregnancies) following Trainee PoCUS.
Vyas [70] 2018	**X**	**X**	Post-training OSCE practical assessment.	Blinded expert review of trainee images over following 12 months for image quality ability to identify OB pathology.	**X**	Patient outcomes.
Wanjiku [21] 2018	3 month post course evaluation.	Online test following self-directed online course- 90% pass mark for eligibility for 1 day practical training. Post course practical assessment.	Post course OSCE/practical assessment with image quality scores. Follow-up in-facility testing scheduled 3–4 months after initial training.	**X**	Frequency & application of PoCUS following training.	**X**
Westerway [50] 2019	Course evaluation- satisfaction, engagement, understanding & relevance of learning.	Identical pre & post course knowledge test repeated at 6 & 11 months follow-up.	Post training practical assessment, repeated at 6 & 11 month follow-up.	**X**	Scanning on returning to clinic/work (confidence and application).	**X**
Abbreviations *KEF – Kirkpatrick Evaluation Framework OSCE- Objective Structured Clinical Examination PoCUS- Point-of-Care UltrasoundUS- Ultrasound						

Fourteen studies performed knowledge assessment of the trainees. Nine of these conducted pre- and post-training tests, and of these, five administered identical exams before and after training. Utilising the same exam allows for a quantifiable measure of trainee improvement but can introduce bias. This risk may be mitigated by randomising question order, not informing trainees the test would be re-administered or discussing test results. Trainees from five studies were required to pass a written test following online self-directed learning before proceeding to practical training. Pre-course learning and testing saves face-to-face time for hands-on learning and can ensure trainees have similar base knowledge on course entry. Six studies reported performing follow-up (knowledge retention) assessment in addition to any immediate post-course testing. Practical assessments or Objective Structured Clinical Examinations (OSCE) were conducted in 16 of the studies, of which half performed consecutive testing allowing for improvement measures. Overall, half the studies performed expert image review (remotely or during training) for quality assurance and to assess competence, and for some, to guide feedback and the necessity for refresher training. This is a useful competence measure where direct supervision is not possible, as it may be performed asynchronously and remotely. Written or practical assessments were not described by seven of the studies. Of these, expert image review was performed by four studies as an indirect evaluation method.

Almost half the studies investigated patient outcomes, several going further to ascertain if PoCUS changed the patient diagnosis and if this impacted their management/treatment. The clinical application was evaluated in nine studies, and in some cases, the frequency of scanning following training was used as an evaluation measure [21,24,30,51,53,62,66,67]. Other measures useful for quality control and course improvement are post-course evaluation surveys, conducted by seven [21,29,45,50,51,54,55] of the reviewed studies, and self-reported post-course scanning confidence that was evaluated by three [45,54,56]. Several studies [48,63] used ‘time to complete scans’ as an evaluation measure. In Vinayak et al. [63] scan times halved after 30 completed examinations with consistent image quality. While not the best measure of competence, speed is an important consideration in time poor, resource-limited settings where extensive scanning times could be prohibitive to PoCUS examination during antenatal consultation. Shah’s et al. [48] study demonstrated that an entire focussed ultrasound assessment (fetal heart rate, head position and estimated gestational age) could be completed by trainees in under 5 minutes, important when facing time-sensitive decisions in an emergency caesarean delivery.

With the aim of revising policy and training to align with the credentialing requirements of their site/country, Lathrop et al. [58] investigated the introduction of a learner portfolio (documented evidence of didactic learning, teaching resources, logged cases and images) and evaluation rubric to demonstrate competence. This approach is consistent with other accrediting authorities’ requirements of scanning logs and evidence of completing a pre-set number of studies. Lathrop et al. [58] was the only study whose participants all progressed to formal accreditation in the use of antenatal PoCUS.

It should be noted many of the reviewed studies were outreach projects that aimed to maximise training opportunities in low-resource settings. While minimal trainee assessment may have been undertaken by some, it is possible that the methods reported were not the only means of assuring trainee competence. Ideally, some form of competence assessment should be performed and support options provided before trainees perform unsupervised clinical scanning. All studies concluded positively regarding the PoCUS training intervention investigated. The outcomes investigated by each study and their main findings are summarised in **Table 3**.

## Barriers to PoCUS following training

Longer-term follow-up training and skills assessments, essential to building confidence and ensuring competence and retention of learning [36], was lacking in over half of the reviewed studies. Insufficient onsite supervising experts on return to clinical practice was also a recurrent theme [21,29,51,56,58,66,67]. This problem is further compounded in some locations by poor telecommunications access, which impedes off-site assistance [22,51,66]. In such cases, telehealth, which is emerging strongly in the wake of the COVID pandemic, would be ineffective as a tool for real-time support of trainees. Several articles in this review offered off-site asynchronous expert image review for quality assurance and feedback [26,45–47,51]. This solution is inadequate in cases where technical hands-on correction is required or immediate image review is needed to guide patient management in an emergency.

Another common barrier included access to quality ultrasound equipment following training. Henwood et al. [53] reported some trainees did not have routine access to ultrasound and the ability to save images from completed examinations, and in Westerway [50], not all trainees scanned patients on return to work due to no or poor/faulty equipment. Busy departments allowing little time for scanning or supervision was another reported barrier. Half of the midwives surveyed in Kimberly et al. [62] reported difficulty finding time to perform ultrasound due to heavy clinical workloads and raised concerns over neglecting other clinical obligations. Almost half of the participants surveyed by Shokoohi et al. [51] listed ‘*lack of time to scan’* as the main perceived challenge integrating PoCUS into patient care.

## Limitations and quality of the reviewed studies

There was a distinct lack of pedagogy described by the reviewed studies. Only Westerway [50] provided a detailed discussion describing the New World Kirkpatrick training evaluation model. Elements of pedagogy were present in other studies’ designs. For example, a constructivist, flipped classroom approach was utilised by a number of training programs [22,51,52,63], but limited description of the principles and foundations of this model were provided.

Generally, the studies suffered from limitations and biases inherent in research conducted in remote settings, including small study designs- low participant numbers, convenience samples, and loss to follow-up. A lack of longer-term follow-up of trainee outcomes was a reported limitation of many of the reviewed studies [46,49,62,66,67]. This is likely the result of geographic isolation, finite funding and an overburdened and transient health workforce [68,69]. Of those that did follow trainee progress, comparison of participants was confounded for some by inconsistent ultrasound exposure on their return to different workplaces, with varying onsite assistance and supervision between sites. Eleven of the studies performed expert review of trainee images, most asynchronously with the expert unable to perform concurrent scanning to verify trainees’ findings [49,58,63]. Blinded image review was specified by several authors [45,70] to mitigate this shortcoming. Poor and worsening participant compliance with patient data recording and image logs was reported by several studies [62,66], some basing image review and quality control on scans selected for uploading by the trainees, potentially biasing results [49].

**Figure 3.**
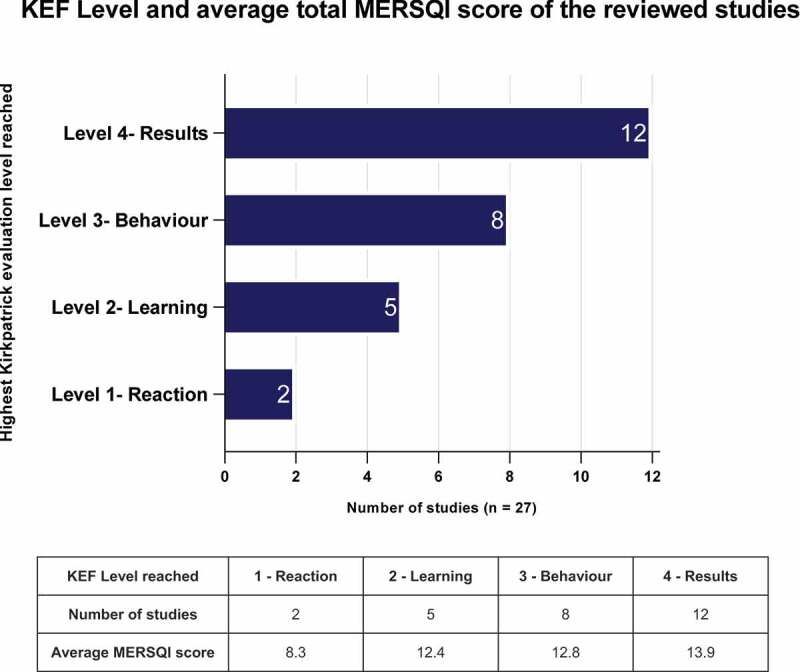
Kirkpatrick evaluation level reached by the reviewed studies.

The overall quality of studies and their evidence varied, with the MERSQI assessment scores ranging from 7.5 to 18 out of 21 (mean score of 12.9). The review lacked randomised, controlled studies necessary to achieve a top MERSQI score. The upper KEF levels 3 and 4 were reached by 3/4s of the included studies. Studies reaching KEF levels 1–2 had an average MERSQI score of 11.2 and KEF Levels 3–4 averaged 13.5. Figure 3 illustrates the KEF level reached by the included studies and corresponding average MERSQI score. **Table A5** of the **Appendix** presents the MERSQI evaluation of each reviewed study inclusive of corresponding KEF level and colour coded bias assessment ranking. The limited use or reporting of conceptual frameworks and models underpinning the development of the educational programs reviewed is reflected in the generally low MERSQI validity scores and underpinning bias ratings; categories commonly underreported in medical education research [71,72]. However, it should be noted that ‘limited reporting’ increases the risk of bias but does not necessarily mean the educational development is of poor quality [72].

## Discussion

This review identified 27 observational studies of moderate to low quality within the specified eligibility criteria despite an inclusive search strategy (obstetric or obstetric inclusive PoCUS training of professionals from any healthcare discipline globally). Description of theoretical concepts or pedagogy underpinning the training programs was generally lacking. Substantial heterogeneity in training formats was found between the reviewed studies, and half lacked follow-up support, training and assessment important to the safe ongoing practice of PoCUS (see **Table A4- Training methods and delivery**). The studies’ findings were generally positive, reporting improved knowledge (where pre- and post-assessments were conducted), and competence being attained despite the substantial variation in course durations (3 hours to several years). Variation in competence assessment and levels of evidence was also observed (see **Table 4- Trainee and course evaluation**), with 11 of the 27 studies not surpassing KEF levels 1 or 2 of assessing immediate reaction to training and knowledge gained. This is a recognised trend in which medical education researchers commonly cease evaluation at the lower KEF levels, finding longer-term investigations required for evaluations at levels 3 and 4 difficult to accomplish [73,74]. This is especially the case for PoCUS training research, which is predominantly conducted in low-resource settings where additional logistical challenges exist.

We identified several recently published systematic reviews of PoCUS training [36,75–77]. All were limited to the instruction of medical physicians, excluding allied health professionals. Other differences included setting, with Rajamani et al. [36] (2020) and Andersen et al. [76] (2019) focusing on general practice and critical care, respectively. Dickson et al. [77] (2017) included studies of trainees with no prior formal ultrasound training only. Consistent with this review’s findings, variation in teaching curriculum and assessment methods, and an overall low standard of study quality were unanimously reported.

Rajamani et al. [36] reported a distinct lack of high-quality evidence on PoCUS competence, with two-thirds of the reviewed studies failing to describe important details on how assessments were conducted, and very few utilising bias minimisation strategies important to observational study designs [78]. None of the 42 studies identified by Rajamani et al. performed follow-up repeat assessments essential to assessing learning retention and safety to practice, and most of the educational programs reviewed failed to follow recommended processes for assessing PoCUS competence.

Anderson et al. [76] found a ‘*great variety of pedagogic approaches*’ and substantial disparity in training durations (2 to 320 hours) between their included studies, which were reported to be of ‘*low quality… mainly because of issues with design and reporting*’. Assessment methods also varied but focussed PoCUS scans were found to require less training. Higher diagnostic accuracy and frequency of clinical use was also reported for obstetric indications, consistent with the findings in this review. These outcomes, and the need to use non-ionising imaging modalities in pregnancy highlight the utility of antenatal PoCUS in the hands of appropriately trained healthcare providers.

### Strengths and limitations of this review

This literature review has relevance to medical educators, researchers, clinicians and policymakers interested in developing curriculum and translating PoCUS safely into clinical practice. To the authors’ knowledge, this is the first published systematic review of worldwide antenatal PoCUS training of multidisciplinary healthcare clinicians. It provides comprehensive tables summarising the reviewed studies PoCUS teaching and evaluation methods. The variable training and evaluation methods described and limitation in reporting made direct comparison of study results for metanalysis unfeasible, meaning effective and ineffective training approaches could not be confidently discerned. Whether one approach to training and assessment was superior to another could not be reliably established, limiting this review. Such heterogeneity, while complicating synthesis of evidence, can offer the advantage of examining the consistency of findings and generalizability of interventions across studies, assessing the relative feasibility of different educational approaches [71,79]. While the level of evidence and detail in reporting was lacking in many of the reviewed studies, a strength of this review is evident in the identification of this gap and the onus for more comprehensive and comparable longer-term studies with which to establish a stronger evidence base for antenatal PoCUS. An inclusive search string with broad criteria ensured a wide cross-section of antenatal PoCUS studies could be scrutinised. However, only two peer reviewed medical databases (MEDLINE and EMBASE) were searched and ‘English only’ articles included. Published studies from non-English-speaking countries in particular would have been missed.

### Recommendations for future research and practice

Robust clinical studies demonstrating the efficacy of training models, and the clinical impact of trainee operated antenatal PoCUS on quality of care and maternal/fetal outcomes are needed. Economic analyses investigating the cost-effectiveness of PoCUS training and implementation would be valuable to justify and inform future programs.

The use of Telehealth with antenatal PoCUS for real-time scanning assistance (Teleultrasound) in the clinical setting also merits further investigation, along with Artificial Intelligence systems that have the capacity to assist minimally trained operators in unsupervised clinical environments [80]. Advancements in Teleultrasound systems now make it possible for the remote clinician to view the ultrasound monitor, images and probe position, communicate via live video and text message with the operator, and even take control of the ultrasound machines functions and demonstrate findings to the patient, all in real time [81]. Many of the reviewed studies are unlikely to have the resources and infrastructure (quality internet/broadband) to support such advanced systems, but they do offer considerable advantages for remote supervision of ultrasound trainees and may see greater utilisation in the future as the technology becomes more affordable.

Only one article in this review reported on the patient experience. Vinayak et al. [63] surveyed patients, reporting they felt trainee performed PoCUS during antenatal care was safe, convenient & reassuring, providing a better antenatal visit experience, increased confidence in care delivery and increased spouse attendance. Considering the propensity for cultural minorities to live in remote communities and the underutilisation of Antenatal care services in these regions [82–84], investigating the patient and partners’ perspective on trainee provided antenatal ultrasound would be beneficial to inform curriculum development with an aim to providing culturally sensitive patient centred care.

## Conclusion

PoCUS is an increasingly utilised diagnostic tool that can enhance the physical exam and guide clinical decision making. It has particular utility in rural clinical practice and developing countries, where advancements in ultrasound equipment and telemedicine are opening new avenues for its establishment. Its lack of regulation and rapid expansion into most specialties underscores the need to establish standards in PoCUS training, competency and on-the-job scanning, ensuring providers are safe to practice. Acknowledging the urgent need for these skills and the difficulty accessing training in remote areas, regulation must be implemented carefully to preserve the time and financial advantages offered by PoCUS training.

Quality education programs require careful and informed consideration in their initial course design, with ongoing review of curricula, training resources, knowledge/practical skills assessment, availability of expert trainers, follow-up support and evaluation. Overall, findings from this review support intensive PoCUS training courses for task-shifting and upskilling of the medical and allied health workforce. However, significant heterogeneity in training, evaluation and research methodologies in the included studies was observed. Quality longitudinal studies with comparable evidence are needed to help frame policy guidelines and inform validated antenatal PoCUS training programs, ensuring the safe implementation of this valuable healthcare resource.

## Data Availability

Additional data referred to but not included in the main manuscript is provided in the Appendix as supplementary material (‘Additional Tables’).

## Systematic review registration

This systematic review is registered with the International Prospective Register of Systematic Reviews (PROSPERO registration number CRD42021230267).

## APPENDIX

**Table A1.** Search strategy

	Search terms 1 Pregnancy/Antenatal	Search terms 2 Ultrasound	Search term 3 Point-of-Care/Bedside	Search terms 4 Education/Training	
**MEDLINEMESH HEADINGS**	Obstetrics/ Pregnancy/ Prenatal care/ Prenatal diagnosis/ Pregnancy complications/ Pregnancy outcome/	Ultrasound/ Ultrasonography/ Prenatal/ Echography/ Diagnostic imaging/	Point of care systems/	Education/ Clinical education/ Continuing education/ Education program/ Health education/ Medical education/ Nursing education/ Postgraduate education/ Allied health education/ idwifery education/ Outcome of education/	Nurse midwifery education/ Continuing education provider/ Training/ Simulation training/ Staff training/ High fidelity simulation training/ In service training/ Evaluation study/ Program evaluation/ Teaching/
**EMBASE MESH HEADINGS**	Obstetrics/ Pregnancy/ Pregnancy complications/ Pregnancy outcome/	Ultrasound/ Echography/ Diagnostic imaging/	Point-of-care testing/	Education/ Clinical education/ Continuing Education/ Education program/ Health Education/ Medical education/ Nursing education/ Postgraduate education/ Allied health education/ idwifery education/Outcome of education/	Nurse midwifery education/ Continuing education provider/ Training/ Simulation training/ Staff training/ High fidelity simulation training/ In service training/ Evaluation study/ Program evaluation/ Teaching/
**KEYWORDS**	**Perinatal** **Prenatal** **Antenatal** **Pregn*** (Pregnant, Pregnancy, Pregnancies) **Obstetr*** (Obstetric, Obstetrics, Obstetrician)	**Ultraso*** (Ultrasound, Ultrasonography, Ultrasonic)	**‘Point of care’** **‘Point-of-care’** **POCUS** **Bedside**	**Evaluat*** (Evaluation, evaluating, evaluate) **Educat*** (Education, Educational, Educate, Educating) **Train*** (Train, Training, Trainer, Trainee) **Workshop*** (Workshop, Workshops) **Skill*** (Skill, Skills)	
**SEARCH STRING EMBASE**	Obstetrics/ or Pregnancy/ or Pregnancy complications/ or Pregnancy outcome/ AND **(Perinatal or Prenatal or Antenatal or Pregn* or Obstetr*).mp** AND Ultrasound/ or Echography/ or Diagnostic imaging/ AND **(Ultraso*).mp** AND Point-of-care systems/ AND **(‘Point of care’ or ‘Point-of-care’ or POCUS or Bedside).mp** AND Education/ or Clinical education/ or Continuing education/ or Education program/ or Health education/ or Medical education/ or Nursing education/ or Postgraduate education/ or Allied health education/ midwifery education/ or Outcome of education/or Nurse midwifery education/ or Continuing education provider/ or Training/ or Simulation training/ or Staff training/ or High fidelity simulation training/ or In service training/ or Evaluation study/ or Program evaluation/ or Teaching/ AND **(Evaluat* or Educat* or Train* or Workshop* or Skill*).mp**				
**SEARCH STRING MEDLINE**	Obstetrics/ or Pregnancy/ or Prenatal care/ or Prenatal diagnosis/ or Pregnancy complications/ or Pregnancy outcome/ AND **(Perinatal or Prenatal or Antenatal or Pregn* or Obstetr*).mp** AND Ultrasound/ or Ultrasonography, Prenatal/ or Echography/ or Diagnostic imaging/ AND **(Ultraso*).mp** AND Point-of-care testing/ AND **(‘Point of care’ or ‘Point-of-care’ or POCUS or Bedside).mp** AND Education/ or Clinical education/ or Continuing education/ or Education program/ or Health education/ or Medical education/ or Nursing education/ or Postgraduate education/ or Allied health education/ midwifery education/ or Outcome of education/or Nurse midwifery education/ or Continuing education provider/ or Training/ or Simulation training/ or Staff training/ or High fidelity simulation training/ or In service training/ or Evaluation study/ or Program evaluation/ or Teaching/ AND **(Evaluat* or Educat* or Train* or Workshop* or Skill*).mp**				
**SEARCH TERMS GOOGLE SCHOLAR**	Antenatal OR obstetric AND PoCUS OR ‘Point of care ultrasound’ OR ultrasound AND training OR education				
**LIMIT**	No limit to

**Table A2.** **Modified Medical Education Research Study Quality Instrument (MERSQI)** [41,42]

Domain	MERSQI Item	Item Score	Max Score
Study design	Single group cross‐sectional or single group post-test only	1	3
	Single group pre-test & post-test	1.5	
	Nonrandomized, 2 groups	2	
	Randomized controlled trial	3	
Sampling	*†Number of Institutions*:		3
	1	0.5	
	2	1	
	3	1.5	
	*Number of trainee/learners		
	1–10	0.5	
	11–50	1	
	51+	1.5	
*Follow-up of training	Not performed	0	3
	Post training/ongoing support	1	
	Follow-up assessment- retention of learning	2	
	Follow-up/refresher training	3	
Type of data	Assessment by participants	1	3
	Objective measurement	3	
Validity assessment of evaluation instrument	*Internal structure:*		3
	Not applicable		
	Not reported	0	
	Reported	1	
	*Content:*		
	Not applicable		
	Not reported	0	
	Reported	1	
	*Relationships to other variables:*		
	Not applicable		
	Not reported	0	
	Reported	1	
Data analysis	*Appropriateness of analysis:*		3
	Inappropriate for study design or type of data	0	
	Appropriate for study design, type of data	1	
	*Complexity of analysis:*		
	Descriptive analysis only	1	
	Beyond descriptive analysis	2	
Outcomes	Satisfaction, attitudes, perceptions, opinions, general facts	1	3
	Knowledge, skills	1.5	
	Behaviours	2	
	Patient/health care outcome	3	
Total possible score			21

Adapted from the **Medical Education Research Study Quality Instrument (MERSQI)** [41,42]. *Categories added- Number of trainees (score of 0.5 to 1.5); Follow-up of training (score of 0 to 3).Categories omitted- Response rate (%). Possible score range from 5 to 21 (original MERSQI instrument 5 to 18).

**Table A3.** **Risk of bias assessment tool** [72]^.^

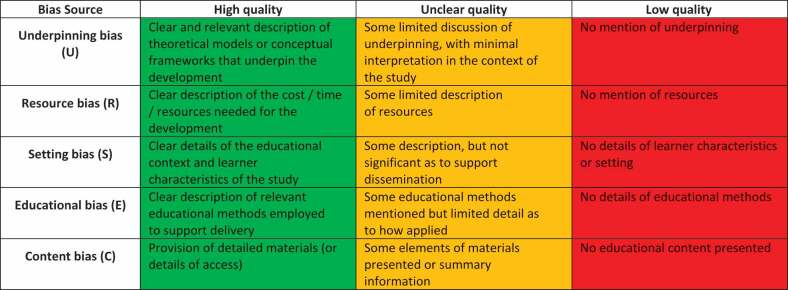

Note- ‘Poor/limited reporting’ increases the risk of bias and does not necessarily mean the educational development is of poor quality. Low quality limits utility for readers in determining transferability of the educational development [72].

**Table A4.** Training methods and delivery

**First author Year Published Location Rural / Urban Income classification**	**Training duration & delivery**	**Trainees** (Number, role/discipline, previous US experience)	**Instructors** (Qualification of instructor/s & ratio of instructor to trainees)	**Curriculum Course topics & Practical skills taught** (Obstetric only or multiple organs systems)	**Follow-up training & support** (Refresher training, follow-up assessment, distance or online support & mentoring provided/offered)
Adler [66] 2008 Africa, Tanzania (refugee camp) Rural LMIC	4 days- Morning lectures followed by afternoon practical.	Multidisciplinary 10 trainees- 4 Physicians, 6 Clinical officers. US experience not specified/unclear.	Instructor credentials and group sizes not specified.	Multiple systems- Basic US physics, knobology, & reviews of clinical US including: **US evaluation of 1^st^ trimester pregnancy; Pregnancy dating & fetal position**; FAST; US evaluation of the abdominal aorta; Hepatobiliary US; US-guided procedures; Soft tissue US; Basic echocardiography; Renal US.	Follow-up at 2 years- 1 instructional session to reinforce prior learning.
Baltarowich [61] 2009 Africa- Sub-Saharan (Training conducted in USA) LIC/LMIC	3 months- didactic lectures, case review sessions, informal teaching sessions, conferences, hands-on sessions using a variety of equipment, & clinical observation rotations.	12 Physicians- 11 radiologists, 1 intern.US experience- 2 to 20+ years.	Training groups of 6. 1:6	Multiple systems- **Focus on OBs with In-depth training in: US physics, instrumentation, cross-sectional anatomy, abdomen, OB/GYN**. Overview of diagnostic US in the areas of vascular, echocardiography, musculoskeletal, paediatrics, thyroid, prostate, sonomammography, & interventional techniques. Care & maintenance of US equipment, preparation techniques for effective teaching.	Follow-up written test after 6 months, 8/12 trainees returned for a 4 week update program at 12 months.
Bell [22] 2016 Africa, Kenya Rural LMIC	1 day & 1–2 follow-up sessions offered every 3–5 months for 1 year.	Multidisciplinary 81 trainees- Equal proportion of doctors, nurses & clinical officers. Several sonographers & 1 lab assistant. Most with no US experience.	US credentialed EM physicians. 4–5 trainees in practical sessions. Ratio 1:4–5	Multiple systems- Physics & machine use; **OB exam for intrauterine pregnancy, cul-de-sac fluid, FH activity & position**; The abdominal, pleural & cardiac assessment for free fluid; The thoracic exam for pneumothorax.	1–2 follow-up refresher sessions offered at 3 to 5 months.
Dalmacion [25] 2018 Asia, Philippines Rural & urban study sites LMIC	2–3 days- Classroom lectures & hands-on training. 2–3 days scanning pregnant women recruited by the study.	Multidisciplinary 20 trainees- 6 doctors, 9 midwives, 5 nurses. US experience not specified/unclear.	Credentialed OB sonographers. All lectures delivered by team leader.	Obstetrics only- Trained to identify & measure 5 OB US images- 1. Viability of the fetus 2. Number of pregnancies 3. Fetal presentation 4. Placental location 5. Amniotic fluid volume (AFV) using maximal vertical pocket	*NR
Dornhofer [54] 2020 Indonesia, East Java Urban UMIC	4 weeks- 6 sessions (3–4 days apart), 6 hours of lectures & 18 hours of practical.	Multidisciplinary 51 Trainees- 19 physicians, 19 midwives, 13 nurses. 36 (65%) had no prior US exposure, 40 (73%) observed US in practice, 4 (7%) attended prior US course.	8 Medical students- 1st year of medical education completed including- lectures on PoCUS for 8 organ systems & 10 hours of supervised hands-on training per organ system, assessment using healthy volunteers. Ratio 1:4 or 1:5 for hands-on training, 2 medical student instructors per US machine/station to 10 trainees.	Multiple systems- 6 session, 1 OB/GYN focussed 1. Basic US including US physics, machine knobology, probe manipulation, & scanning techniques. 2. Pulmonary US, evaluation of pneumothorax, pleural effusions, & pneumonia. 3. Cardiac US in the parasternal long axis view, parasternal short axis view, apical four chamber view, & subxiphoid view. 4. Abdominal US- hepatic, biliary, renal, intestinal, aorta, & inferior vena cava. **5. OB/GYN US including identification of** **intrauterine pregnancy, estimated GA, visualization of the ovaries, & bladder**. 6. Combination of previous sessions to aid FAST & the Focused Assessment with Sonography for HIV-Associated Tuberculosis (FASH).	*NR
Henwood [53] 2017 Africa, Rwanda Rural LIC	10-day course. 6 months scanning assessment for image accuracy.	17 Physicians- US experience not specified/unclear.	EM physicians with expertise in PoCUS, consultant obstetricians & radiologists.	Multiple systems- Introduction to PoCUS & common applications. **OBs- Gestational sac, Yolk sac, Fetal pole, FHR, Endomyometrial mantle, CRL, BPD, FL, Fetal lie, placental location, Placenta previa, Hydramnios**. Echocardiography, Abdominal, Lung, Spleen, Liver, Biliary, Renal, Soft tissue.	Follow-up training every 6 weeks for 6 months trial period with regular practical assessments. Onsite image review & technical assistance.
Kimberly [62] 2010 Africa, Zambia Rural LMIC	6 months- 3 training periods of 2 to 3 weeks interspersed by 2 to 3 months of independent scanning under minimal supervision	21 Midwives- No prior US experience.	3 emergency US fellowship trained physicians.	Obstetrics only- US machine use, cleaning & storage, Patient data entry, image saving (for review), & exam datasheet completion. Practical- Identify basic OB applications: Fetal presentation (vertex or breach), FHR (presence & use of callipers to calculate the rate), Placental location (anterior, posterior, low-lying, or previa), number of gestations & GA based on BPD & FL.	1 year follow-up visit questionnaire of PoCUS application & frequency, no additional training.
Kolbe [26] 2015 Central America, Nicaragua Rural & urban study sites LMIC	Daily morning didactic sessions & practical workshops, afternoon US of patients from the community (course duration unclear), followed by 3 months remote guidance & telecommunication.	Multidisciplinary 4 trainees- 2 postgraduate rural medicine physicians, 1 nurse, 1 nursing assistant. US experience not specified/unclear.	Experienced international sonographers. Ratio 1:4.	Multiple systems- **OBs- Normal pregnancy, Fetal viability; Complicated pregnancy (Ectopic pregnancy, Placenta previa, Placenta abruption)**. Other systems- Echocardiography, Hemodynamic evaluation, Pleural, Lung, Abdominal & Vascular US.	Weekly 60–90 min remote guidance sessions & telecommunication supportfor 3 months following training.
Kotagal [56] 2015 USA, Washington Urban aimed at rural deployment HIC	14 hours- 7 sessions of 2 hours over 3 months.	16 General surgery residents-Limited previous US training (guidance for central venous catheter placement & a brief FAST training).	Multidisciplinary faculty (Surgery, Radiology, Anaesthetics & EM) with expertise in PoCUS.	Multiple systems- Topics- US physics, knobology, safety & artifacts; FAST; Skin & soft tissue US; **OB US (early & late pregnancy)**; Focused echocardiography & volume assessment; Procedural guidance US (paracentesis, thoracentesis, & pericardiocentesis); Vascular US (abdominal aorta & deep vein thrombosis); US guided regional anaesthesia.	*NR
Lathrop [58] 2011 USA, Indianapolis Urban HIC	Half day workshop- 45 minute instruction session followed by practical.	4 Advanced practice Nurses (APN)- 3 attended previous didactic US training.	Credentialed US provider. Ratio 1: 2–3 for practical workstations.	Obstetric Only- Instruction session (45 mins)- Review US standards of care, basic review of US physics & instrumentation, indications, clinical significance & documentation. Practical session- Fetal number, demonstration of life, estimate amniotic fluid, placenta location & fetal presentation. Learning portfolio- (a) documentation of didactic learning (certificates from US-related training or workshops attended); (b) readings (journal articles, clinical bulletins, & practice statements); (c) US cases.	No follow-up of trainees reported but an additional future workshop planned to include more advanced skills, such as fetal biometry, first-trimester US, & cervical length measurement.
Lee [55] 2017 Indonesia, Bandung Urban UMIC	4 weeks – 6 sessions. Each session- 30 min lecture followed by 2 hours hands-on training.	41 Physicians- 22 (53.7%) no prior US experience, 18 (43.9%) observed US in practice, 1 (2.4%) attended prior US course.	Medical students- 1st year of medical education completed including- lectures on PoCUS for 8 organ systems & 10 hours of supervised hands-on training per organ system, assessment using healthy volunteers.	Multiple systems- 6 sessions, 38 US milestones. 1- Basic US including physics, knobology, probe manipulation & scanning techniques. 2- Pulmonary US, evaluation of pneumothorax, pleural effusions & pneumonia. 3- Cardiac US in the parasternal long axis view, parasternal short axis view, apical four chamber view & subxiphoid view. 4- abdominal US (hepatic, biliary, renal, intestinal, aorta & inferior vena cava). 5**- OB/GYN US (identification of intrauterine pregnancy, estimated GA, visualization of the ovaries & bladder)**. 6- combination of previous sessions to aid FAST & the Focused Assessment with Sonography for HIV-Associated Tuberculosis (FASH).	Follow-up scheduled for 12 months to re-test trainees & assess use of US.
Lindgaard [52] 2017 Denmark, Odense Urban HIC	2 days practical & feedback session following online learning package.	5 General Practitioners- 4 specialist GPs, 1 final year resident. No formal US training.	Instructors from Center of Clinical US, University of Aarhus.	Multiple systems- E-learning online lectures- Basic US knowledge- physics & US equipment; Focused US examinations of thoracic structures, abdomen, vascular & musculoskeletal. Practical exams conducted**- Intrauterine pregnancy; GA;** Gall-stones; Ascites; Abdominal aorta.	*NR
Mandavia [67] 2000 USA, California Urban HIC	16 hours over 2 days. 8 hours lectures & 8 hours practical.	Multidisciplinary 80 trainees- 16 attending physicians, 53 ED house officers, 4 physician assistants, 7 medical students. Prior US training experience- none (58), minimal 1–5hrs (6), moderate 5–10 hrs (9), & extensive >10 hrs (7).	Board-certified US trained EM physicians & certified registered diagnostic medical sonographers. Ratio 4:1 per US/station for hands-on learning.	Multiple systems- Curriculum based on Society for Academic Emergency Medicine guidelines. Lectures- Basic physics; **Pelvis & OB**; Right upper quadrant; Renal; Aorta; Trauma; Echocardiography. Practical- OB skills included: **Detection of early intrauterine pregnancy &/or free pelvic fluid; Detection of live fetus in 2^nd^/3^rd^ trimesters**.	Follow-up identical test at 10 months & images performed over this time reviewed for accuracy.
Nathan [49] 2017 5 sites: Democratic Republic of Congo, Karawa Guatemala, Chimaltenango Kenya, Eldoret Pakistan, KarachI Zambia, Lusaka Rural LIC/LMIC/UMIC	2 weeks- One third didactic sessions, two thirds practical scanning. 2 supervised US exams on each day of the course.	Multidisciplinary 41 Trainees- 10 medical officers, 18 nurses, 6 midwives, 7 radiographers. No prior US experience.	Training under the direction of a Radiologist. Local sonographer for ongoing training, QA & trainee evaluation. Image review by: Radiologist (25 yrs in OB US), 2 professors of OBs; a professor of radiology (10 yrs in OB US); & 2 lead OB sonographers.Training groups of 4 to 12 – ratio not specified.	Obstetric only- Curriculum development- Didactic & hands-on instruction, printed materials, extensive quality assurance activities, educational outreach visits, daily schedule, speaker notes for PowerPoint slides, & explicit instructions for each practical scanning session. Topics- Patient communication, safety, & comfort; Infection prevention & control; Care & security of the equipment; US physics & instrumentation; Anatomic planes & introduction to tomography. Practical skills- Detection of cardiac activity; Fetal number, Fetal position, Amniotic fluid assessment; BPD; HC; AC; FL; Placental position; Cervical length at 16–24 weeks; Detection of some anomalies (ventriculomegaly, anencephaly, hydronephrosis, & spina bifida).	For the 12 week pilot period- Monthly follow-up & practical assessment. Local instructors met trainees to discuss performance & observe scanning progress bi-weekly. Image feedback from quality control website. Targeted remedial training given to some trainees.
Rominger [24] 2018 Mexico, Chiapas Rural UMIC	16 days training over 12 month duration. Total of 4 sessions (each session 4 day course) every 3–4 months.	10 Physicians-Little to no prior US experience.	Obstetrician, Paediatric EM physician, EM physician & residents. 6 to 10 trainees per 2 instructors. Ratio 1:3–5	Multiple systems- 1- Introduction to US, equipment use & care, FAST, kidney & bladder evaluation, **basic OBs**. 2- Lung, skin & soft tissue, & musculoskeletal US. **3- OB/GYN physician taught focused on OBs**. 4- Review of US basics, cardiovascular, advanced abdomen, & ocular.	Case logs & images (35%) reviewed for quality assurance & feedback.
Shah [45] 2020 Africa, Uganda LIC	2 days intensive training course followed supervised (Master trainer) scanning 2–3 days per week for 3 weeks.	Multidisciplinary 25 Trainees- 20 midwives, 3 nurses, 2 physicians. No prior US experience.	Certified sonographer, OB/GYN resident physician, family physician & 3 EM physicians with US training. Instructors had prior experience in PoCUS in LMIC. Ratio 1:3 for practical scanning.	Obstetrics only- Short lectures & hands-on demonstrations using healthy volunteers, hands-on live scanning practice on 3^rd^ trimester antenatal patients not in labor, & mock active labor patients. Topics- US knobs & Physics; US Safety; Principles of adult learning; US teaching methods; Hands-on training techniques; Fetal position; Placental position; Fluid volume; Multiple gestation; FHR; Estimating GA: BPD, HC, FL, TCD; Common learner mistakes.	Twice weekly distance/online communication for 3 months.Confidence survey repeated at 3 months. Key informant interviews assessed trainee’ perception of training program
Shah [48] 2014 USA, North-western Urban HIC	3 hours- 1 hour lecture, 2 hours practical.	31 Emergency physicians- 17 attendings, 14 residents. Range of no prior experience to experienced.	US fellowship trained EM physicians. 1:1 for hands-on instruction.	Obstetric only- Mechanics of TA & TV US. General review of 1st trimester US including- Normal US findings in 1st trimester, Other abnormal US findings in early pregnancy (fetal demise, molar pregnancy), finding FHR using M-mode, measurement of GA using CRL, & ectopic pregnancy. Late pregnancy US including fetal lie & identification of the fetal head, & fetal biometry including measurement of BPD, FL, HC & AC. Practical- live models in the 2nd & 3rd trimester of pregnancy.	*NR
Shah [47] 2009 Africa, Rwanda Kirehe & Rwinkwavu in Eastern Province Rural LIC	9 weeks- 1 hour lecture (15 lectures total) followed by 1–2 hours practical.	10 Physicians-Little to no prior US experience.	US training sessions conducted by a 4th year EM resident with prior US experience (including OB US) during residency training & credentialing certification.	Multiple systems- Principles of US physics; Machine operation; Potential uses of US; Outline of lecture series, training expectations, image & data sheet recording instructions. **OB US including 1^st^ trimester basics- ectopic or molar pregnancy, estimating GA, & evaluation of fetal position, cervix & placenta**. Cardiac US; Hepato-biliary US; Renal US; Advanced applications- US evaluation of DVT, procedures including vascular access, abscess evaluation & drainage, US guided paracentesis & thoracentesis.	Image accuracy & quality assessed by expert review. Telecommunication (email) support offered.
Shah [46] 2008 Africa, Rwanda, Kirehe & Rwinkwavu in Eastern Province Rural LIC	9 weeks- 1 hour lecture (15 lectures total) followed by 1–2 hours practical.	15 physicians- 0–4 h of prior US training solely in OBs.	US training sessions conducted by a 4th year EM resident with prior US experience (including OB US) during residency training & credentialing certification. Cardiac echo lecture given by visiting Cardiologist.1:1 for scanning practice during ward rounds. Other group sizes not specified.	Multiple systems- Principles of US physics; Machine operation; Potential uses of US; Outline of lecture series, training expectations, image & data sheet recording instructions. **OB US including 1^st^ trimester basics- ectopic or molar pregnancy, estimating GA, & evaluation of fetal position, cervix & placenta**. Cardiac US; Hepato-biliary US; Renal US; Advanced applications- US evaluation of DVT, procedures including vascular access, abscess evaluation & drainage, US guided paracentesis & thoracentesis.	Image accuracy & quality to be assessed by expert review (Shah 2009). Telecommunication (email) support offered.
Shaw-Battista [29] 2015 USA, California Urban HIC	10 asynchronous online modules followed by 4 hours training- 2 hour seminar, 2 hour practical.	Multidisciplinary Initial course- 72 trainees- Midwives, nurses, family practice physicians, physician assistants Developed course- 162 trainees- Midwives, nurses, nurse practitioners, medical & midwifery students, family practice & OB physicians, physician assistants. Range of no prior US experience to experienced.	Senior OB & family medicine residents (advanced trainees & licensed clinicians).Ratio 1:3–5 during practical sessions.	Obstetrics only- Asynchronous online module topics- Indications for OB US; Documentation, communication, liability, & risk-reduction strategies; US physics, equipment, & optimizing image quality; Normal 1st trimester US; Abnormal 1st trimester US; Limited 2nd & 3rd trimester US; Fetal biophysical profile; 2nd & 3rd trimester fetal biometrics; Use of OB US on the labor & delivery ward; Interprofessional OB US training & practice.	*NR
Shokoohi [51] 2019 Africa, Tanzania, Malawi & Uganda Rural & urban LIC/LMIC	2 day training course. Online modules & educational materials provided.	Multidisciplinary 49 Trainees (initial 63 with 78% response rate)- 7 midwives, 42 physicians (different specialties).21/49 never performed independent US,29/49 limited US training during residency.	Sonographer. Ratio 1:3–4 during course. Ratio 1:1 for in clinic training.	Multiple systems- Topics- review of the concepts for clinician- performed US, introduction to the US machine & controls, the focused assessment with sonography for trauma/focused assessment with sonography for human immunodeficiency virus/tuberculosis (FAST/FASH), abdominal US exam, echocardiography, lung, renal/bladder & abdominal US **Midwives & obstetricians received focused training on gynaecologic US & 1^st^ & 3^rd^ trimester US.**	Telecommunication support- Online feedback on transmitted images from independent scanning after training
Stolz [30] 2015 Africa, Uganda LIC	2 years- US lectures delivered intermittently over the 2-year ‘emergency care for non-physicians’ curriculum (train the trainer model) with the core US lectures occurring early in the programme.	13 Nurses-US experience not specified/unclear.	EM physicians with fellowship training in emergency US or strong clinical competency in emergency US.	Multiple systems- 1. Introduction to PoCUS, Machine care, basic physics & US principles, image optimisation, knobology, safety, patient consent & documentation in the medical record. 2. FAST; 3. Echocardiography; 4. Hepatobiliary US; 5. Renal US; 6. Soft tissue US; 7. Lung US; 8. Aorta US; 9. Inferior vena cava US; 10. **OB/GYN US- Evaluation of intrauterine pregnancy, signs of ectopic pregnancy, pelvic free fluid, adnexal mass, foetal heart rate, foetal movement, fetal dating & fetal presentation** 11. Abdominal US; 12. Procedural guidance with US; 13. US assessment for deep vein thrombosis.	*NR
Swanson [85] 2014 Africa-Uganda Rural LIC	6 weeks- Lectures, small-group tutorials, audio-visual materials & supervised clinical scanning.	14 midwives- 13 no prior US experience, 1 experienced.	Primary instructors’ credentials not specified. Sonographer for ongoing scanning support.	Obstetric only- Curriculum included US physics, relevant anatomy & physiology, instrumentation & basic maintenance. Screening included- cardiac activity (yes, no), fetal presentation (cephalic, breech & others) & fetal number, Placental position (normal, previa & low lying), Pain & bleeding (complete abortion, incomplete abortion, blighted ovum, ectopic pregnancy or placental abnormality).	Ongoing training resource in onsite sonographer available to trainees on return to clinic
Vinayak [63] 2017 Africa, Kenya Outer regional & Rural LMIC	4 weeks- 1 hour lecture, 6 hours practical, 1 hour feedback & debrief per day (days per week unclear). Several days of assessment after course completion.	3 midwives- No prior US experience.	‘Experienced sonographers. Ratio 1:3.	Obstetrics only- 1. E-learning module- introduction to general principles of US, US physics & OB US. 2. Post module test 3. Didactic lectures- US knobology, general & OB US 4. Hands-on practical experience. Practical skills- HC, AC, FL & FHR & rhythm. Placenta location & distance of lower lip from the cervix & estimated the amount of amniotic fluid (obtained from four images, one image for each quadrant).	*NR
Vyas [70] 2018 America, Panama (Training in USA) Rural HIC	12 hours- 6 sessions on OB US instruction, termed “Rural Obstetric Ultrasound Triage Exam” (ROUTE).	8 medical students- Prior basic US training in medical school curriculum.	Trainer- 3rd year OB/GYN resident physician. Assessment by board certified OB/GYN physicians.	Obstetric only- ROUTE including identification of- fetal lie, placental position, DVP, amniotic fluid index (AFI), BPD, & HC for 2nd & 3rd trimester.	*NR
Wanjiku [21] 2018 Africa, Kenya Rural LMIC	1 day practical training following self-directed online pre-training.	Multidisciplinary 33 trainees- Clinical officers, Nurses, Medical officers. All had previously completed a novel pilot PoCUS training program. Other prior US training excluded participation.	Certified Instructors.	Multiple systems- Standardized OSCE trainees assessed on – FAST (Trauma assessment), 1^st^ & 2^nd^/3^rd^ trimester scanning skills, i.e., identification of the gestational sac, 1^st^ trimester dating using crown-rump length, detecting & measuring the FHR, identifying the presenting part, locating the placenta, & measurement of HC & BPD.	Follow-up in-facility testing scheduled 3–4 months after initial training. 3–4 month refresher training offered to trainees who failed practical assessment.
Westerway [50] 2019 Australia Rural & urban HIC Indonesia/Central Asia/Timor Leste UMIC/LMIC	6 separate PoCUS courses compared. Ranging from 4 to 18 hours total. Up to 6 hours lecture, 3–12 hours practical.	Multidisciplinary 55 trainees- 15 medical practitioners, 23 midwives, 9 nurses, 8 radiographers. No prior US experience.	Experienced OB US tutors. Same lead lecturer for all 6 courses. Ratio from 1: 2–5 (average 1:4).	Obstetric only- Curriculum- Clinical history- pregnancy progression; Knobology, image optimisation (depth, focus, gain) & probe use; Theory behind performing a limited fetal anatomical assessment & 3rd trimester fetal biometry. Practical checklist- Image optimisation; Probe movements; Fetal lie; FHR; Recognition of fetal anatomy (heart, stomach & bladder); Amniotic fluid estimate (single deepest pocket); Identification of cervix & placental position; Fetal biometry measurements (BPD, HC, AC, FL).	Follow-up test & practical at 6 & 11 months
Key- *NR = not reported/described					
**Abbreviations** AC – Abdominal Circumference AFV – Amniotic fluid volume BPD – Biparietal Diameter CRL – Crown to Rump Length DVP – Deepest Vertical Pocket ED – Emergency Department EGA – Estimated Gestational Age EM – Emergency Medicine FAST – Focused Assessment with Sonography in Trauma		FH – Fetal Heart FHR – Fetal Heart Rate FL – Femur Length GA – Gestational Age HC – Head Circumference HIC – High-income country LIC – Low-income country LMIC – Lower-middle-income country LRS – Low-resource setting		OB – Obstetric OB/GYN – Obstetrics and Gynaecology OSCE – Objective Structured Clinical Examination TA – Transabdominal TCD – Trans Cerebellar Diameter TV – Transvaginal UMIC – Upper-middle-income country US – Ultrasound

**Table A5.** **Quality assessment and bias ranking of included articles using a modified Medical Education Research Study Quality Instrument (MERSQI)** [41,42]

## References

[1] Campbell S. A short history of sonography in obstetrics and gynaecology. Facts Views Vis Obgyn. 2013;5(3):213–36. PMCID: PMC3987368. 24753947 PMC3987368

[2] Australian Department of Health. *Clinical Practice Guidelines: pregnancy care 2019 edition*. Canberra: Australian Government, 2019, viewed 11 May 2021. <https://www.health.gov.au/sites/default/files/pregnancy-care-guidelines_0.pdf>.

[3] Moore CL, and Copel JA. Point-of-care ultrasonography. N Engl J Med. 2011;364(8):749–757. 21345104 10.1056/NEJMra0909487

[4] Nicolson M. Imaging and imagining the fetus: the development of obstetric ultrasound. Baltimore: Johns Hopkins University Press; 2013.

[5] World Health Organization (WHO). *WHO recommendations on antenatal care for a positive pregnancy*. 2016, World Health Organization, Geneva, viewed 25 march 2020, <https://www.who.int/publications/i/item/9789241549912>.

[6] Whitworth M, Bricker L, and Mullan C. Ultrasound for fetal assessment in early pregnancy. Cochrane Database Syst Rev. 2015;7:1–57. DOI:10.1002/14651858.CD007058.pub3. PMC6464767 26171896

[7] Wiafe YA, Odoi AT, and Dassah ET. The Role of Obstetric Ultrasound in Reducing Maternal and Perinatal Mortality. In: Minin IV, and Minn OV, editors. Ultrasound imaging - medical applications. London: IntechOpen; 2011.

[8] Murugan VA, Murphy BOS, Dupuis C, et al. Role of ultrasound in the evaluation of first-trimester pregnancies in the acute setting. Ultrasonography. 2020;39(2):178–189. DOI:10.14366/usg.19043. 32036643 PMC7065984

[9] Salomon LJ, Alfirevic Z, Da Silva Costa F, et al. ISUOG practice guidelines: ultrasound assessment of fetal biometry and growth. Ultrasound Obstetrics Gynecol. 2019;53(6):715. DOI:10.1002/uog.20272. 31169958

[10] Community Affairs References Committee *Availability and accessibility of diagnostic imaging equipment around Australia*. Australian Parliament House, Canberra, 2018, viewed 20 March 2020, <https://www.aph.gov.au/Parliamentary_Business/Committees/Senate/Community_Affairs/Diagnosticimaging/Report>.

[11] Hofmeyr GJ, Haws RA, and Bergström S, et al. Obstetric care in low-resource settings: what, who, and how to overcome challenges to scale up? Int J Gynaecol Obstet. 2009;107 Suppl 1:S21-44, s44-25 DOI:10.1016/j.ijgo.2009.07.017. 19815204

[12] McClure EM, Nathan RO, Saleem S, et al. First look: a cluster-randomized trial of ultrasound to improve pregnancy outcomes in low income country settings. BMC Pregnancy Childbirth. 2014;14(1):73. DOI:10.1186/1471-2393-14-73. 24533878 PMC3996090

[13] World Health Organization (WHO). *Maternal mortality*. 2020, World Health Organization, viewed 25 March 2021, <https://www.who.int/news-room/fact-sheets/detail/maternal-mortality>.

[14] World Health Organization (WHO). *Newborns: improving survival and well-being*. 2020, World Health Organization, viewed 9 April 2021, <https://www.who.int/news-room/fact-sheets/detail/newborns-reducing-mortality>.

[15] Shah S, Bellows BA, Adedipe AA, et al. Perceived barriers in the use of ultrasound in developing countries. Crit Ultrasound J. 2015;7(1):1–5. DOI:10.1186/s13089-015-0028-2. 26123609 PMC4485671

[16] Australasian Society of Ultrasound in Medicine (ASUM). Minimum education & training requirements for ultrasound practitioners. Australas J Ultrasound Med. 2017;20(3):132–135. DOI:10.1002/ajum.12061. 34760485 PMC8409892

[17] Fentress M, Heyne TF, Barron KR, et al. Point-of-care ultrasound in resource-limited settings: common applications. South Med J. 2018;111(7):424–433. DOI:10.14423/SMJ.0000000000000827. 29978229

[18] Australasian Society of Ultrasound in Medicine (ASUM). *Discussion paper: definition of Point of Care Ultrasound (POCUS*), The Australasian Society of Ultrasound in Medicine (ASUM), viewed 28 March 2020, <https://www.asum.com.au/files/public/RealTime/2017/ASUM-Discussion-Paper-Definition-of-POCUS.PDF>.

[19] Australian Sonographer Accreditation Registry (ASAR). Sonographer accreditation. 2020, Australian Sonographer Accreditation Registry (ASAR), viewed 25 March 2020, <https://www.asar.com.au/sonographer-info/accredited-medical-sonographer/>.

[20] Australasian Sonographers Association (ASAR). The Australasian sonographers association 2019–20 Australian government pre-budget submission. 2020, The Australasian Sonographers Association, Melbourne, viewed 24 March 2020, <https://www.sonographers.org/news-item/1997/the-asa-2019-20-australian-government-pre-budget-submission>.

[21] Wanjiku GW, Bell G, and Wachira B. Assessing a novel point-of-care ultrasound training program for rural healthcare providers in Kenya. BMC Health Serv Res. 2018;18(1):607. < 10.1186/s12913-018-3196-5>. 30081880 PMC6091199

[22] Bell G, Wachira B, and Denning G. A pilot training program for point-of-care ultrasound in Kenya. Afr J Emergency Med. 2016;6(3):132–137. DOI:10.1016/j.afjem.2016.03.002. PMC6234186 30456079

[23] Vinayak S, and Brownie S. Collaborative task-sharing to enhance the Point-Of-Care Ultrasound (POCUS) access among expectant women in Kenya: the role of midwife sonographers. J Interprof Care. 2018;32(5):641–644. < 10.1080/13561820.2018.1470499>. 29746179

[24] Rominger AH, Gomez GAA, Elliott P. The implementation of a longitudinal POCUS curriculum for physicians working at rural outpatient clinics in Chiapas, Mexico. Crit Ultrasound J. 2018;10(1). DOI:10.1186/s13089-018-0101-8 PMC6092270 30109455

[25] Dalmacion GV, Reyles RT, Habana AE, et al. Handheld ultrasound to avert maternal and neonatal deaths in 2 regions of the Philippines: an iBuntis intervention study. BMC Pregnancy Childbirth. 2018;18(1):32. DOI:10.1186/s12884-018-1658-8. 29347926 PMC5774122

[26] Kolbe N, Killu K, Coba V, et al. Point of care ultrasound (POCUS) telemedicine project in rural Nicaragua and its impact on patient management. J Ultrasound. 2015;18(2):179–185. DOI:10.1007/s40477-014-0126-1. 26191106 PMC4504858

[27] Smith A, Parsons M, Renouf T, et al. A mixed-methods evaluation of a multidisciplinary point of care ultrasound program. Med Teach. 2019;41(2):223–228. DOI:10.1080/0142159X.2018.1461202. 29688110

[28] Hoppmann RA, Rao VV, Poston MB, et al. An integrated ultrasound curriculum (iUSC) for medical students: 4-year experience. Crit Ultrasound J. 2011;3(1):1–12. DOI:10.1007/s13089-011-0052-9. 21516137 PMC3064888

[29] Shaw-Battista J, Young-Lin N, Bearman S, et al. Interprofessional obstetric ultrasound education: successful development of online learning modules; case-based seminars; and skills labs for registered and advanced practice nurses, midwives, physicians, and trainees. J Midwifery Women’s Health. 2015;60(6):727–734. DOI:10.1111/jmwh.12395. 26769384

[30] Stolz LA, Muruganandan KM, Bisanzo MC, et al. Point-of-care ultrasound education for non-physician clinicians in a resource-limited emergency department. Trop Med Int Health. 2015;20(8):1067–1072. DOI:10.1111/tmi.12511. 25808431

[31] Australasian Society of Ultrasound in Medicine (ASUM). A guide to providing an ultrasound workshop. 2016, Australasian Society of Ultrasound in Medicine (ASUM), viewed 11 May 2021, <https://www.google.com/search?client=firefox-b-d&q=A+Guide+to+Providing+an+Ultrasound+Workshop#>.

[32] Australasian Society of Ultrasound in Medicine (ASUM). Guidelines for course providers seeking CCPU course accreditation. 2015, Australasian Society of Ultrasound in Medicine (ASUM), viewed 6 May 2021, <https://www.google.com/search?client=firefox-b-d&q=Guidelines+for+Course+Providers+Seeking+CCPU+Course+Accreditation>.

[33] Kumar A, Kugler J, and Jensen T. Evaluation of trainee competency with Point-of-Care Ultrasonography (POCUS): a conceptual framework and review of existing assessments. J Gen Intern Med. 2019;34(6):1025–1031. DOI:10.1007/s11606-019-04945-4. 30924088 PMC6544692

[34] Leonardi M, Murji A, and D’Souza R. Ultrasound curricula in obstetrics and gynecology training programs. Ultrasound Obstet Gynecol. 2018;52(2):147–150. DOI:10.1002/uog.18978. 29205571

[35] Wright J, Noriega O, and Ho H. The application of hand-held ultrasound scanner in teaching of telemedicine and rural medicine. 2014;8(1):87–91. DOI:10.5005/jp-journals-10009-1340.

[36] Rajamani A, Shetty K, Parmar J, et al. Longitudinal Competence Programs for Basic Point-of-Care Ultrasound in Critical Care: a Systematic Review. Chest. 2020;158(3):1079–1089. DOI:10.1016/j.chest.2020.03.071. 32343964

[37] Collins K, Collins C, and Kothari A. Point-of-care ultrasound in obstetrics. 2019;22(1):32–39. DOI:10.1002/ajum.12133. PMC8411729 34760534

[38] Torloni MR, Vedmedovska N, Merialdi M, et al. Safety of ultrasonography in pregnancy: WHO systematic review of the literature and meta-analysis. Ultrasound Obstetrics Gynecol. 2009;33(5):599–608. DOI:10.1002/uog.6328. 19291813

[39] Abramowicz JS. Benefits and risks of ultrasound in pregnancy. Semin Perinatol. 2013;37(5):295–300. < 10.1053/j.semperi.2013.06.004>. 24176149

[40] Miller DL. Safety assurance in obstetrical ultrasound. Semin Ultrasound CT MR. 2008;29(2):156–164. DOI:10.1053/j.sult.2007.12.003. 18450141 PMC2390856

[41] Reed DA, Cook DA, and Beckman TJ, et al. Association between funding and quality of published medical education research. JAMA. 2007;298(9):1002–1009. DOI:10.1001/jama.298.9.1002. 17785645

[42] Reed DA, Beckman TJ, Wright SM, et al. Predictive validity evidence for medical education research study quality instrument scores: quality of submissions to jgim’s medical education special issue. J Gen Intern Med. 2008;23(7):903–907. DOI:10.1007/s11606-008-0664-3. 18612715 PMC2517948

[43] Kirkpatrick DL. Techniques for evaluating training programes. 1979;33:78. ISSN 0041-0861.

[44] Kirkpatrick DL. Evaluating training programs the four levels/Donald L. Kirkpatrick, James D. Kirkpatrick. San Francisco CA: Berrett-Koehler; 2006.

[45] Shah S, Santos N, Kisa R, et al. Efficacy of an ultrasound training program for nurse midwives to assess high-risk conditions at labor triage in rural Uganda. PLoS ONE. 2020;15(6):e0235269. DOI:10.1371/journal.pone.0235269. 32603339 PMC7326214

[46] Shah S, Noble VE, Umulisa I, et al. Development of an ultrasound training curriculum in a limited resource international setting: successes and challenges of ultrasound training in rural Rwanda. Int J Emerg Med. 2008;1(3):193–196. DOI:10.1007/s12245-008-0053-z. 19384515 PMC2657276

[47] Shah SP, Epino H, Bukhman G, et al. Impact of the introduction of ultrasound services in a limited resource setting: rural Rwanda 2008.(Research article)(Report). BMC Int Health Hum Rights. 2009;9(1):4. DOI:10.1186/1472-698X-9-4. 19327157 PMC2667437

[48] Shah S, Adedipe A, Ruffatto B, et al. BE-SAFE: bedside sonography for assessment of the fetus in emergencies: educational intervention for late-pregnancy obstetric ultrasound. West J Emerg Med. 2014;15(6):636–640. DOI:10.5811/westjem.2014.7.18480. 25247032 PMC4162718

[49] Nathan RO, Swanson JO, and Swanson DL, et al. Evaluation of focused obstetric ultrasound examinations by health care personnel in the democratic Republic Of Congo, Guatemala, Kenya, Pakistan, and Zambia. Curr Probl Diagn Radiol. 2017;46(3):210–215. DOI:10.1067/j.cpradiol.2016.11.001. 28057388 PMC5413583

[50] Westerway SC. Comparing the effectiveness of training course formats for point-of-care ultrasound in the third trimester of pregnancy. Australas J Ultrasound Med. 2019;22 1:45–50. DOI:10.1002/ajum.12125. 34760536 PMC8411680

[51] Shokoohi H, Raymond A, Fleming K, et al. Assessment of point-of-care ultrasound training for clinical educators in Malawi, Tanzania and Uganda. Ultrasound Med Biol. 2019;45(6):1351–1357. DOI:10.1016/j.ultrasmedbio.2019.01.019. 30904246

[52] Lindgaard K, and Riisgaard L. Validation of ultrasound examinations performed by general practitioners. Scand J Prim Health Care. 2017;35(3):256–261. < 10.1080/02813432.2017.1358437>. 28776457 PMC5592352

[53] Henwood PC, Mackenzie DC, Liteplo AS, et al. Point-of-care ultrasound use, accuracy, and impact on clinical decision making in Rwanda Hospitals. J Ultrasound Med. 2017;36(6):1189–1194. DOI:10.7863/ultra.16.05073. 28258591

[54] Dornhofer K, Farhat A, and Guan K, et al. Evaluation of a point-of-care ultrasound curriculum taught by medical students for physicians, nurses, and midwives in rural Indonesia. J Clin Ultrasound. 2020;48(3):145–151. DOI:10.1002/jcu.22809. 31876301

[55] Lee JB, Tse C, Keown T, et al. Evaluation of a point of care ultrasound curriculum for Indonesian physicians taught by first-year medical students. World J Emerg Med. 2017;8(4):281–286. DOI:10.5847/wjem.j.1920-8642.2017.04.006. 29123606 PMC5675969

[56] Kotagal M, Quiroga E, Ruffatto BJ, et al. Impact of point-of-care ultrasound training on surgical residents’ confidence. J Surg Educ. 2015;72(4):e82–e87. DOI:10.1016/j.jsurg.2015.01.021. 25911457 PMC4786300

[57] World Health Organization (WHO). WHO recommendations on antenatal care for a positive pregnancy experience: ultrasound examination: highlights and key messages from the World Health Organization’s 2016 global recommendations. 2018, World Health Organization, viewed 20 April 2021, <https://apps.who.int/iris/bitstream/handle/10665/259946/WHO-RHR-18.01-eng.pdf;jsessionid=DDAF84D516491D79FAD4E8AC33222AFD?sequence=1>.

[58] Lathrop A, and Blackburn M. Learner portfolios and hands-on workshop to facilitate and evaluate nurses’ learning in obstetric ultrasound. J Obstet Gynecol Neonatal Nurs. 2011;40(5):654–661. DOI:10.1111/j.1552-6909.2011.01287.x. 22273422

[59] Engum SA, and Jeffries PR. Interdisciplinary collisions: bringing healthcare professionals together. Collegian. 2012;19(3):145–151. < 10.1016/j.colegn.2012.05.005>. 23101349

[60] American College of Emergency Physicians (ACEP). Ultrasound guidelines: emergency, point-of-care and clinical ultrasound guidelines in medicine. Ann Emerg Med. 2016;69:e27–e54. DOI:10.1016/j.annemergmed.2016.08.457. 28442101

[61] Baltarowich OH, Goldberg BB, and Wilkes AN, et al. Effectiveness of ‘teaching the teachers‘ initiative for ultrasound training in Africa. Acad Radiol. 2009;16(6):758–762. DOI:10.1016/j.acra.2008.12.023. 19362026

[62] Kimberly HHM A:, Mennicke M:, and Liteplo A:, et al. Focused maternal ultrasound by midwives in rural Zambia. Ultrasound Med Biol. 2010;36(8):1267–1272. < 10.1016/j.ultrasmedbio.2010.05.017>. 20691916

[63] Vinayak S, Sande J, Nisenbaum H, et al. Training midwives to perform basic obstetric point-of-care ultrasound in rural areas using a tablet platform and mobile phone transmission technology-A WFUMB COE project. Ultrasound Med Biol. 2017;43(10):2125–2132. DOI:10.1016/j.ultrasmedbio.2017.05.024. 28716434

[64] Chalouhi GEB, Ville Y, and Ville Y. Ultrasound simulators in obstetrics and gynecology: state of the art. Ultrasound Obstetrics Gynecol. 2015;46(3):255–263. DOI:10.1002/uog.14707. 25346451

[65] Tolsgaard MG. Assessment and learning of ultrasound skills in obstetrics & gynecology. Dan Med J. 2018;65(2, B5445 29393042). 29393042

[66] Adler D, Mgalula K, Price D, et al. Introduction of a portable ultrasound unit into the health services of the Lugufu refugee camp, Kigoma District, Tanzania. Int J Emerg Med. 2008;1(4):261–266. DOI:10.1007/s12245-008-0074-7. 19384640 PMC2657264

[67] Mandavia DP, Aragona J, Chan L, et al. Ultrasound training for emergency physicians–a prospective study. Acad Emerg Med. 2000;7(9):1008–1014. DOI:10.1111/j.1553-2712.2000.tb02092.x. 11043996

[68] Willis-Shattuck M, Bidwell P, Thomas S, et al. Motivation and retention of health workers in developing countries: a systematic review. BMC Health Serv Res. 2008;8(1):247. DOI:10.1186/1472-6963-8-247. 19055827 PMC2612662

[69] Siriwardhana C. Promotion and reporting of research from resource-limited settings. Infect Dis (Auckl). 2015;8:25–29. DOI:10.4137/IDRT.S16195. 26396528 PMC4562664

[70] Vyas A, Moran K, Livingston J, et al. Feasibility study of minimally trained medical students using the Rural Obstetrical Ultrasound Triage Exam (ROUTE) in rural Panama. World J Emerg Med. 2018;9(3):216–222. DOI:10.5847/wjem.j.1920-8642.2018.03.009. 29796147 PMC5962457

[71] Reed D, Price EG, Windish DM, et al. Challenges in systematic reviews of educational intervention studies. Ann Intern Med. 2005;142(12_Part_2):1080–1089. DOI:10.7326/0003-4819-142-12_Part_2-200506211-00008. 15968033

[72] Gordon M, Patricio M, Horne L, et al. Developments in medical education in response to the COVID-19 pandemic: a rapid BEME systematic review: BEME Guide No. 63. Med Teach. 2020;42(11):1202–1215. DOI:10.1080/0142159X.2020.1807484. 32847456

[73] Yardley S, and Dornan T. Kirkpatrick’s levels and education evidence. Med Educ. 2012;46(1):97–106. DOI:10.1111/j.1365-2923.2011.04076.x. 22150201

[74] Moreau KA. Has the new Kirkpatrick generation built a better hammer for our evaluation toolbox? Med Teach. 2017;39(9):999–1001. DOI:10.1080/0142159X.2017.1337874. 28649887

[75] Moses A, Weng W, and Orchanian-Cheff A, et al. Tteaching point-of-care ultrasound in medicine: a scoping review. 2020;15(2):13–29. DOI:10.22374/cjgim.v15i2.368.

[76] Andersen CA, Holden S, and Vela J, et al. Point-of-care ultrasound in general practice: a systematic review. Ann Fam Med. 2019;17(1):61–69. DOI:10.1370/afm.2330. 30670398 PMC6342599

[77] Dickson R, Duncanson K, and Shepherd S. The path to ultrasound proficiency: a systematic review of ultrasound education and training programmes for junior medical practitioners. Australas J Ultrasound Med. 2017;20(1):5–17. DOI:10.1002/ajum.12039. 34760465 PMC8409858

[78] Labrecque JA, and Kaufman JS. Commentary: can a Quasi-experimental Design Be a Better Idea than an Experimental One? Epidemiology. 2016;27(4):500–502. DOI:10.1097/EDE.0000000000000485. 27031041

[79] Mulrow C, Langhorne P, and Grimshaw J. Integrating heterogeneous pieces of evidence in systematic reviews. Ann Intern Med. 1997;127(11):989–995. DOI:10.7326/0003-4819-127-11-199712010-00008. 9412305

[80] Abramowicz JS. Obstetric ultrasound: where are we and where are we going? Ultrasonography. 2021;40(1):57–74. DOI:10.14366/usg.20088. 33105529 PMC7758093

[81] Loria K. Remote access: How ultrasound in telemedicine is changing education, training, and patient care. Radiology today magazine. 2021, viewed 6 May 2021, <https://www.radiologytoday.net/archive/rt0818p24.shtml>.

[82] Rurangirwa AA, Mogren I, Nyirazinyoye L, et al. Determinants of poor utilization of antenatal care services among recently delivered women in Rwanda: a population based study. BMC Pregnancy Childbirth. 2017;17(1):142. DOI:10.1186/s12884-017-1328-2. 28506265 PMC5430598

[83] Jacobs C, Michelo C, and Moshabela M. Why do rural women in the most remote and poorest areas of Zambia predominantly attend only one antenatal care visit with a skilled provider? A qualitative inquiry. BMC Health Serv Res. 2018;18(1):409. DOI:10.1186/s12913-018-3212-9. 29871624 PMC5989442

[84] Rumbold AR, Bailie RS, Si D, et al. Delivery of maternal health care in Indigenous primary care services: baseline data for an ongoing quality improvement initiative. BMC Pregnancy Childbirth. 2011;11(1):16. DOI:10.1186/1471-2393-11-16. 21385387 PMC3066246

[85] Swanson JO, Kawooya MG, Swanson DL, et al. The diagnostic impact of limited, screening obstetric ultrasound when performed by midwives in rural Uganda. J Perinatol. 2014;34(7):508–512. DOI:10.1038/jp.2014.54. 24699218

